# Cross talk between RNA modification writers and tumor development as a basis for guiding personalized therapy of gastric cancer

**DOI:** 10.1186/s40246-022-00386-z

**Published:** 2022-04-22

**Authors:** Shi Zhang, Guanghao Kuang, Yao Huang, Xinxin Huang, Weiyu Wang, Guoqiang Wang

**Affiliations:** 1 Department of Gastrointestinal Surgery, The Second Affiliated Hospital of Guangzhou Medical University, 250 Changgang East Road, Haizhu District, Guangzhou, 510260 Guangdong China; 2 Department of Oncology, HaploX Biotechnology, Co., Ltd., Shenzhen, 518057 China

**Keywords:** Gastric cancer, RNA modifications, Writers, Prognosis, Epithelial–mesenchymal transition (EMT), Tumor microenvironment (TME), Bioinformatics analysis

## Abstract

**Background:**

Gastric cancer (GC) shows high metastasis and low survival. RNA modification writers play critical roles in tumor development. This study examined the clinical significance of RNA modification writers in GC prognosis based on four types of adenosine modifications (m^1^A, m^6^A, APA and A-to-I).

**Results:**

Writers demonstrated high mutation and expression in GC patients. Different expressions of 26 RNA modification writers were differentially associated with GC prognosis. High-WM score group appeared worse overall survival, higher immune infiltration and activation of EMT pathways than low-WM score group. WM score was correlated with both miRNAs-targeted signaling pathways and patients’ sensitivity to chemotherapeutic drugs and efficacy of immunotherapy.

**Conclusions:**

This study further revealed the close association between adenosine-related RNA modifications and progression of GC. A cross talk between EMT and RNA modification was identified to be one of the mechanisms underlying GC development. Our WM scoring system could serve as a clinical indicator for predicting GC prognosis. Importantly, the WM score could guide personalized treatments such as chemotherapy and immunotherapy for GC patients.

**Supplementary Information:**

The online version contains supplementary material available at 10.1186/s40246-022-00386-z.

## Background

Gastric cancer (GC) shows a high incidence rate each year worldwide. In 2020, 1,089,103 new GC cases were diagnosed, accounting for about 11.1% of all new cancer cases that year [1]. The incidence and mortality demonstrate regional variations. GC has the highest diagnosis rate in Japanese male population (32.5/100,000) and Mongolian female population (13.2/100,000) [1]. The top 5 regions with a high incidence are Eastern Asia, Eastern Europe, South America, Western Asia and South Europe. In China, the morbidity rate of GC reaches 26.54 per 100,000 in male and 11.09 per 100,000 in female [2]. An estimated number of 478,508 new cases were diagnosed in Chinese population in 2020, with an age-standardized incidence rate (ASR) of 20.6/100,000 [1]. Depending on different stages, the 5-year overall survival (OS) of GC is between 10 and 93% [3]. Even if patients have been properly treated with chemotherapies, the median OS of metastatic GC patients is shorter than 1 year [4]. Thus, an effective indicator predictive of GC prognosis or capable of guiding targeted therapy for GC patients is highly needed.

In the past decades, epigenetic modification has been illustrated to be closely associated with cancer development [5]. A number of studies applied the epigenetic modification in clinical diagnosis or treatment of GC. For example, Meng et al. discovered a strong association between DNA methylation and tumor microenvironment (TME) in GC and explored a DNA methylation score (DMS) that may help guide personalized immunotherapy [6]. Jing et al. explored the association between N^6^-methyladenosine (m^6^A) enzymes and GC prognosis and found that m6A writers (METTL3, RBM15 and WTAP), m^6^A erasers (FTO and ALKBH5) and m6A readers (YTHDF3 and YTHDC2) were related to GC development and prognosis [7]. Based on m^6^A RNA methylation, a prognostic signature composed of FTO, RBM15 and ALKBH5 has been constructed to predict GC overall survival (OS) [8]. A review on m6A modifications summarized that m6A writers, erasers and binding proteins play important roles in gastrointestinal tract cancers [9]. These findings suggested that biomarkers identified based on epigenetic modification could assist clinical decision-making.

Epigenetic modification, especially the most frequent RNA modification, is an essential part of gene regulation. Dysregulation of epigenetic modification can result in aberrant gene expression and dysregulated functional pathways [5, 10, 11]; moreover, dysfunction of methylation signaling pathways will increase or reduce modifications, thereby affecting translation or post-translational processing. It was demonstrated that reduced m^6^A methylation could activate oncogenic Wnt/PI3K-Akt pathway, thereby promoting malignant transformation of GC cells [12]. Zhang et al. identified three m^6^A clusters (cluster A, B and C) corresponding to three immune phenotypes in GC [13]. Specifically, cluster A (immune-excluded phenotype), which is also known as T cell-suppressive type, shows high activity of angiogenesis, epithelial–mesenchymal transition (EMT) and TGF-β pathways [13].

Although m^6^A modification has been found to be closely involved in cancer progression through altering TME or oncogenic pathways [14–18], other less studied but common RNA modifications such as N^1^-methyladenosine (m^1^A), alternative polyadenylation (APA) and adenosine-to-inosine (A-to-I) editing are also critical in cancer development [19]. Zhao et al. first demonstrated that m^1^A enzymes were dysregulated in gastrointestinal cancer and was involved in tumorigenesis possibly via ErbB and mTOR pathways [20]. APA as an essential step of processing mRNA maturation is present in at least 70% of human genes [21]. Evidence revealed that 95% APA genes have shorter 3′-untranslated regions (3′ UTRs) in cancers, which can facilitate tumor cell proliferation through inhibiting microRNAs (miRNAs)-mediated suppression [22, 23]. CPSF6 involved in APA process has been proven to induce tumor cell proliferation and inhibit apoptosis through APA-mediated 3′UTR shorting in gastric cells [24]. A-to-I RNA editing is one of posttranscriptional modifications that is thought to be altered in thousands of miRNA binding sites in 32 cancer types [25]. In GC, ADAR1 and ADAR2, enzymes of A-to-I editing, are dysregulated and its mis-editing is highly responsible for GC pathogenesis [26], suggesting that the activity of ADAR1 and ADAR2 may serve as potential biomarkers for GC.

Based on previous findings, this study elucidated the relation between four major RNA modifications (m^1^A, m^6^A, APA and A-to-I editing) related to adenosine editing and GC through applying bioinformatics analysis. We developed an RNA modification writers-based signature strongly correlated with GC prognosis, TME and EMT pathway. The signature demonstrated strong potentials in guiding chemotherapy and immunotherapy.

## Results

### Different gene mutation patterns and expressions of RNA modification writers in cancer cells and normal cells

**Fig. 1:**
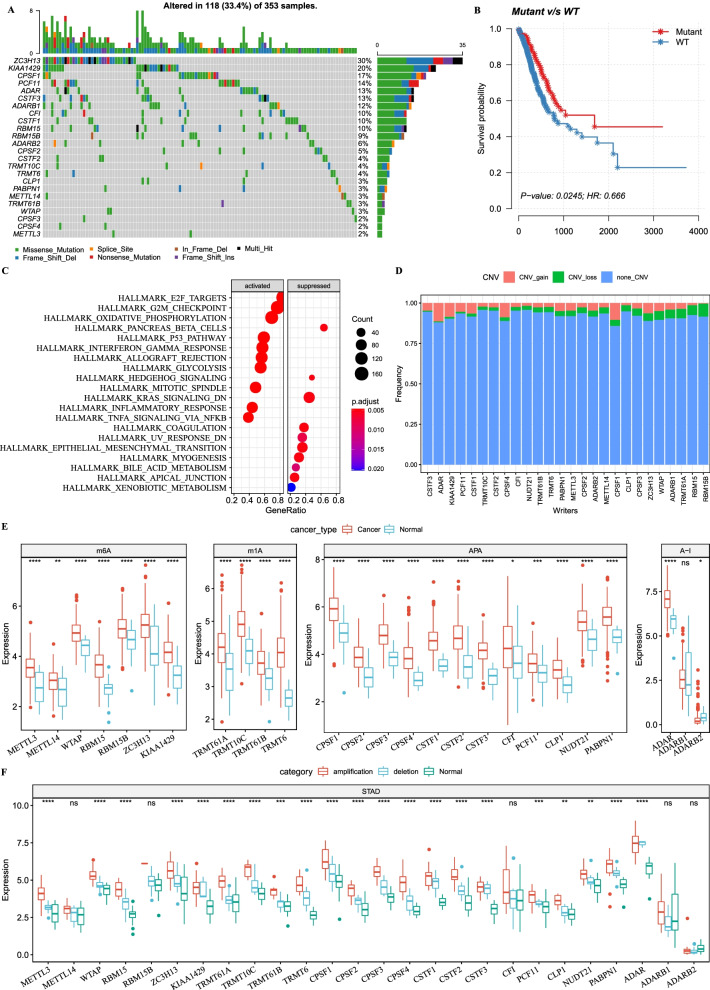
Mutation patterns and expression level of 26 RNA modification writers. **A** The top 20 mutated genes in 118 of 353 samples in TCGA-STAD dataset. Eight types of mutations were presented, including missense, nonsense, splice site mutation, in-frame deletions, frame-shift deletions or insertions, and different combinations of multiple genetic mutations (multi-hit). **B** Kaplan–Meier survival curve of mutant group and wild-type (WT) group. Log-rank test was performed. **C** Enriched pathways of hallmark gene set in mutant group and WT group. The dot size represents the count numbers of genes. **D** CNV distribution of 26 RNA modification writers. Red, green and blue represent gain of copy number, loss of copy number and no CNV, respectively. **E** Comparison of the expression level of 26 writers between cancer samples and normal samples grouped by m^6^A, m^1^A, APA and A-I. Student’s *t* test was performed. **F** The expression level of 26 writers among gain of copy number (amplification), loss of copy number (deletion) and normal samples. Kruskal–Wallis test was performed. *p < 0.05, **p < 0.01, ***p < 0.001, ****p < 0.0001. *ns* no significance

The workflow of this study was shown (Additional file 1: Fig. S1). The study included four types of RNA modifications writers (writers of m^1^A, m^6^A, APA and A-to-I editing) and analyzed a total of 26 genes of writers. In TCGA-STAD dataset, 353 samples were recruited to analyze gene mutations and mutation patterns of the 26 writers, and 118 of them were mutant (Fig. 1A). *ZC2H13* was the most frequent mutated gene, showing a mutation frequency of 30%, and there were 10 genes that reached a mutation frequency up to 10% (Fig. 1A). The majority of mutations were missense mutation and frame-shift deletion. Interestingly, patients with mutations showed a longer OS than those without, suggesting a potential relationship between the writers and GC (*p* = 0.0245, Fig. 1B). GSEA was applied to enrich hallmark gene sets in the mutated samples. Here, we detected a series of tumor-related gene sets, such as E2F targets, Myc targets, G2/M checkpoint, pancreas beta cells and interferon-gamma response (Fig. 1C). In addition, by using PathScore tool, we observed that these significantly mutated genes were highly enriched in BARD1 pathway (56.5% patients, *p* < 1e−16) and interferon alpha–beta signaling pathway (33% patients, *p* = 0.048, Additional file 2: Fig. S2).

In addition to small-scale mutations, copy number variation (CNV) is a major factor contributing to aberrant gene expression. Our result showed that *ADAR*, *CPSF1* and *CPSF4* had high frequency of increased copy numbers, while *RBM15* and *RBM15B* showed a high proportion of decreased copy numbers; interestingly, no CNV was detected in *KIAA1429* (Fig. 1D). Compared with normal samples, GC cancer samples exhibited obviously higher expression of m^6^A, m^1^A and APA writers (Fig. 1E), but ADAR only showed significantly higher expression in 3 A-to-I writers (Fig. 1E). Notably, *RBM15B* with loss of copy numbers still expressed higher in cancer samples. To further understand the relation of CNV and gene expression, the samples were classified into CNV amplification and CNV deletion groups. In both amplification and deletion groups, we observed a higher expression level in most writer genes in GC samples than in normal samples (Fig. 1F). The above results indicated that the expression of writers was involved in a complicated regulation network and may be affected by small- or large-scale mutations. Additionally, these data also suggested that abnormal expression was a potential factor for GC tumorgenesis and development.

### Expression patterns of RNA modification writers were associated with GC prognosis and tumor immune cell infiltration

**Fig. 2:**
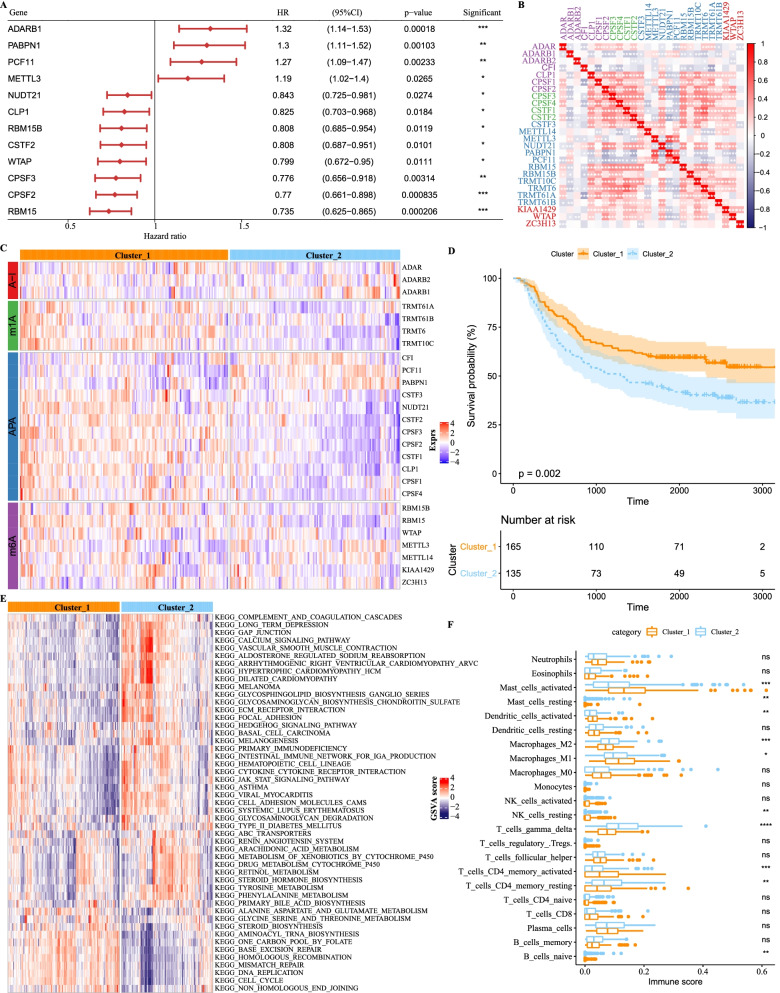
Different expression patterns of RNA modification writers. **A** Univariate Cox regression of 26 writers and 12 writers had a close relation with GC prognosis. **B** Spearman rank correlation analysis of 26 writers. Red means positive correlation and blue means negative correlation. **C** Unsupervised consensus clustering of 300 samples in GSE62254 dataset. Red and violet represent high and low expression, respectively. **D** Kaplan–Meier survival curve of cluster 1 (165 samples) and cluster 2 (135 samples). Log-rank test was performed. **E** Enriched KEGG pathways of two clusters analyzed by GSVA. Red and blue represent high and low GSVA enrichment score, respectively. **F** Enrichment and distribution of 22 immune cells in two clusters. Student *t* test was performed. *p < 0.05, **p < 0.01, ***p < 0.001, ****p < 0.0001. *ns* no significance, *CI* confidence interval, *HR* hazard ratio

To investigate the relation between each writer and GC prognosis, univariate Cox regression analysis was performed on 300 samples of GSE62254 dataset. Twelve out of 26 writers including ADARB1, PABPN1, PCF11 and METTL3 were positively related to hazard ratio (HR) and significantly related to the cancer prognosis, and NUDT21, CLP1, RBM15B, CSTF2, WTAP, CPSF3, CPSF2 and RBM15 were negatively related to HR (Fig. 2A). To validate their prognostic performance, we implemented immunohistochemistry (IHC) of two writers (ADARB1 and RBM15B) on GC tissues. Consistent with the above analysis, ADARB1 was higher expressed and RBM15B was lower expressed in advanced gastric cancer tissue (Additional file 3: Fig. S3), but both the two showed opposite expressions in early GC tissue, demonstrating the reliability of these writers of serving as prognostic indicators. Spearman rank analysis on the correlations among writers revealed interactions among different writers (Fig. 2B).

As the specific roles of writers are different in the development of GC prognosis and interactions among writers, we speculated whether different expression patterns of all writers would lead to different prognostic results. Thus, unsupervised consensus clustering was performed to classify 300 samples into two groups based on different expression patterns of 26 writers (Fig. 2C). Survival analysis showed a significant difference between the two groups, and cluster 1 group tended to develop a more favorable OS than cluster 2 (*p* = 0.002, Fig. 2D). Then, we used GSEA to analyze the correlation between the two clusters and functional pathways. Cluster 1 group was enriched to pathways related to DNA repair and cell cycle, such as base excision repair pathway, homologous recombination pathway, mismatch repair pathway, DNA replication pathway and cell cycle pathway (Fig. 2E). Cluster 2 group presented a lower GSVA score in the above pathways, but many metabolism pathways, for example, arachidonic acid metabolism pathway, and metabolism of xenobiotics were enriched by cytochrome P450 pathway and drug metabolism cytochrome pathway (Fig. 2E). On biological process (BP) and molecular function (MF) terms, the two clusters also exhibited apparently distinct enrichment (Additional file 4: Fig. S4). A total of 1046 (21.0%) and 1770 (35.5%) BP terms were significantly annotated in clusters 1 and 2, respectively (FDR < 0.05). Metabolism and RNA modification-related BP terms were highly enriched in Cluster 1, while Cluster 2 had a high correlation with cell proliferation, migration and autophagy (Additional file 4: Fig. S4A). Moreover, 328 and 251 MF terms were significantly enriched in clusters 1 and 2, respectively (FDR < 0.05); specifically, cluster 1 was more correlated with RNA synthesis, and cluster 2 was more associated with protein binding and extracellular matrix (Additional file 4: Fig. S4B). The results further supported the conclusion that Cluster 2 had worse prognosis, which may be resulted from its high activity of tumor cell proliferation and migration.

Previous studies showed that RNA modifications are correlated with tumor immune infiltration; therefore, we performed CIBERSORT to calculate the enrichment score of immune-related cells and analyzed its correlation with writers. Of the four types of writers, the expression of m^6^A and APA writers, especially gamma delta T cells, activated memory CD4 T cells, M0 macrophages, activated dendritic cells, resting and activated mast cells, were found to be closely associated with immune infiltration (Additional file 5: Fig. S5). Activated mast cells and M1 macrophages were greatly enriched in cluster 1, and gamma delta T cells, M2 macrophages and resting memory CD4 T cells were noticeably enriched in cluster 2 (Fig. 2F). Through using Timer method, we also observed similar results that cluster 2 had higher immune infiltration than cluster 1 (Additional file 6: Fig. S6).

### Construction of a prognostic signature based on genes related to RNA modification writers

**Fig. 3:**
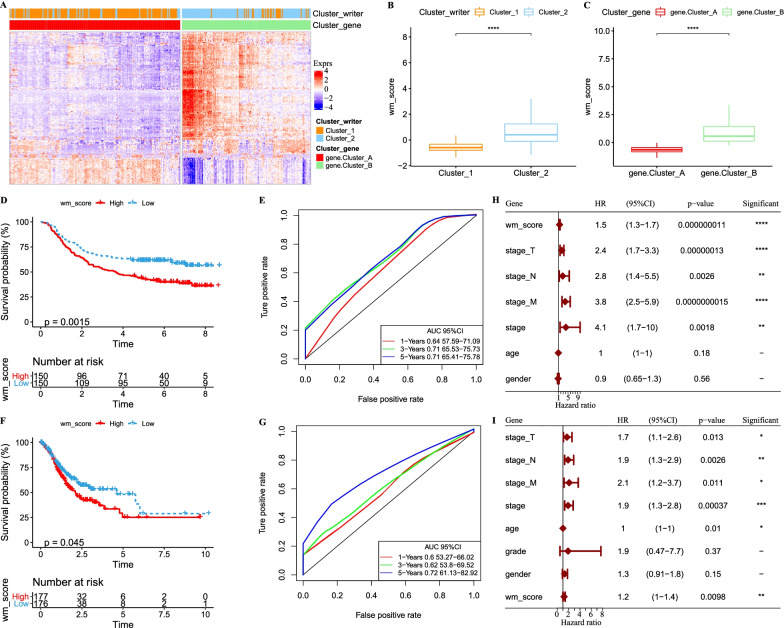
Construction of a signature of RNA modification writers. **A** Unsupervised consensus clustering of 194 RNA modification genes in GSE62254 dataset, and the comparison of two kinds of clustering. **B**, **C** WM score of different RNA modification patterns, cluster 1 and cluster 2 (B), cluster A and cluster B (C). Wilcoxon test was performed. **D** Kaplan–Meier survival curve of high-WM score and low-WM score groups in GSE62254 dataset. Log-rank test was performed. **E** ROC analysis of 1 year, 3 years and 5 years for WM score effectiveness in GSE62254 dataset. **F** Kaplan–Meier survival curve of high-WM score and low-WM score groups in TCGA-STAD dataset. Log-rank test was performed. **G** ROC analysis of 1 year, 3 years and 5 years for WM score effectiveness in TCGA-STAD dataset. (H-I) Multivariate Cox regression analysis of WM score, T stage, N stage, M stage, stage, age and gender in GSE62254 (**H**) and TCGA-STAD (**I**) dataset. Log-rank test was performed. *p < 0.05, **p < 0.01, ***p < 0.001, ****p < 0.0001

Variations in expression patterns of RNA modification writers normally result in different influence on GC prognosis; we therefore developed a signature for predicting the prognosis based on the genes related to RNA modification writers. A total of 194 differentially expressed genes related to RNA modification were identified through univariate Cox regression analysis. Gene ontology (GO) analysis revealed that these 194 genes were involved in multiple biological functions (Additional file 7: Fig. S7A–C). Next, according to unsupervised clustering analysis, 300 samples of GSE62254 dataset were stratified into two groups (cluster A and cluster B) (Additional file 9: Table S1). Survival analysis exhibited significantly differential OS of the two groups, with higher OS in cluster B (*p* = 0.0032, Additional file 7: Fig. S7D). Two types of systems for classifying GC patients were constructed based on 26 writers and 194 genes related to RNA modification writers. The performance of the two systems in clustering 300 samples were similar (Fig. 3A), indicating the reliability of developing a prognostic signature using RNA modification writers.

Based on 194 genes, we established a WM (Writers of RNA Modification) scoring system, which was shown as WM score = *Σ*(coefficient *i*) * (expression *i*) [27]. The scoring system showed robust performance in different groups, in which cluster 2 had higher WM score than cluster 1 (*p* = 3.40e−29, Fig. 3B). Consistently, cluster B also had higher WM score than cluster A (*p* = 9.70e−49, Fig. 3C). Samples in GSE62254 dataset were classified into high-risk and low-risk groups with distinct OS using the WM score (*p* = 0.0015, Fig. 3D). According to receiver operator curve (ROC), WM score showed an area under ROC curve (AUC) of 0.64, 0.71 and 0.71, respectively, in predicting 1-year, 3-year and 5-year OS (Fig. 3E). Similarly, 353 samples in TCGA-STAD dataset were divided into high-risk and low-risk groups with differential prognosis (*p* = 0.045, Fig. 3F), and ROC analysis also demonstrated a robust performance of the WM score (Fig. 3G). Furthermore, multivariate Cox regression analysis in both datasets verified that WM score could serve as an effective indicator for GC prognosis prediction, with a hazard ratio of 1.5 (95% CI = 1.3–1.7, *p* < 0.0001) in TCGA-STAD dataset and 1.2 (95% CI = 1.0–1.4, *p* = 0.0098) in GSE62254 dataset (Fig. 3H, I).

### WM score was correlated with tumor microenvironment

**Fig. 4:**
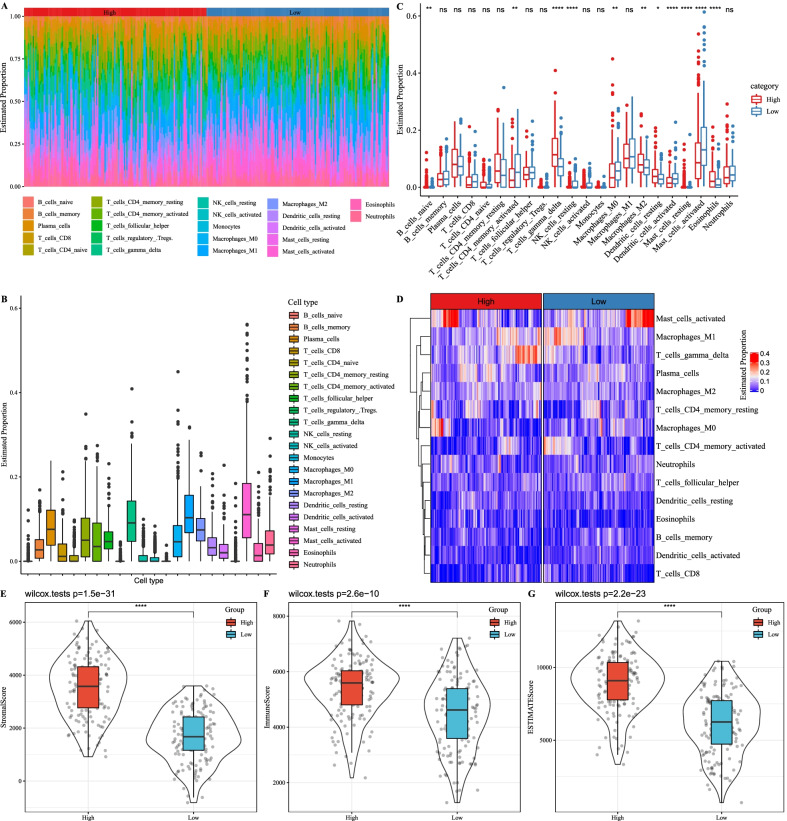
The relation between WM score and immune infiltration. Heatmap (**A**) and box plot (**B**) for describing the distribution and proportion of 22 immune cells. **C** Enrichment of 22 immune cells in high- and low-WM score groups. Student’s *t* test was performed. **D** Proportion of 15 immune cells whose immune score was nonzero in over half samples. **E**–**G** Stromal score, immune score and ESTIMATE score calculated by CIBERSORT in high- and low-WM score groups. Wilcoxon test was performed. *p < 0.05, **p < 0.01, ****p < 0.0001. *ns* no significance

To examine whether WM score was related to tumor microenvironment (TME), CIBERSORT was applied to calculate the proportion and enrichment score of 22 immune cells. In GSE62254 dataset, the proportion of 22 immune cells in high-risk and low-risk groups was shown in the heatmap and histogram (Fig. 4A–C). Among the 22 immune cells, gamma delta T cells, M1 macrophages and activated mast cells consisted of a large proportion (Fig. 4B), and 11 immune cells were differentially enriched in the two groups (Fig. 4C). Particularly, activated memory T cells, M0 macrophages, activated dendritic cells and activated mast cells were more enriched in the low-risk group, while gamma delta T cells, M2 macrophages, resting dendritic cells and eosinophils were more enriched in the high-risk group (*p* < 0.05, Fig. 4C). After excluding seven immune cells without expression from more than half of the samples, 15 immune cells remained and their distributions in the two groups are shown in Fig. 4D. Immune infiltration analysis using CIBERSORT showed a higher enrichment score of stromal score, immune score and ESTIMATE score in high-risk group than low-risk group, indicating that WM score was positively related to immune infiltration (Fig. 4E, F).

### The relation between WM score and clinical features, epithelial–mesenchymal transition

**Fig. 5:**
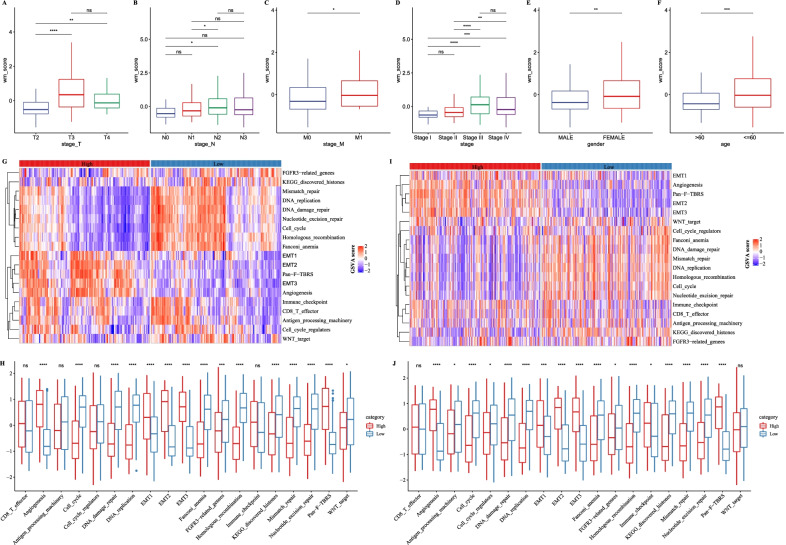
The relation between WM score and clinical features, EMT-related pathways. **A**–**F** The relation between WM score and clinical features including T stage (**A**), N stage (**B**), M stage (**C**), stage I to IV (**D**), gender (**E**) and age (**F**) in GSE62254 dataset. Heatmap (**G**) and box plot (**H**) for describing the relation between WM score and EMT-related pathways in GSE62254 dataset. Heatmap (**I**) and box plot (**J**) for describing the relation between WM score and EMT-related pathways in TCGA-STAD dataset. Wilcoxon test was performed. *p < 0.05, **p < 0.01, ***p < 0.001, ****p < 0.0001. *ns* no significance

Next, we identified the relation between WM score and clinical features including T stage, N stage, M stage, stage I to IV, gender and age. In T stage, T3 had the highest WM score, and the score of T2 was significantly different from that of T3 and T4 (Fig. 5A). Though the difference of WM score was not obviously different in T stage or M stage, there was still a tendency of the higher WM score correlating with the progression of stage (Fig. 5B, C). Moreover, such a tendency was also observed in stage I to IV, which manifested significant differences between stage I and III, stage II and III, stage II and IV (Fig. 5D). Notably, the WM score of female group was higher than male group, and age was another important factor leading to differential WM score (Fig. 5E, F).

As epithelial–mesenchymal transition plays a crucial role in tumor progression, we also assessed the enrichment of EMT-related pathways in high-risk and low-risk groups using GSVA. In GSE62254 dataset, EMT1, EMT2, Pan-F-TBRS, EMT3 and angiogenesis pathways were highly enriched to high-risk group, while DNA replication, DNA repair and cell cycle pathways were more enriched to low-risk group (Fig. 5G, H). The majority of pathways showed significant differences between the two groups. In addition, similar result was also observed in TCGA-STAD dataset (Fig, 5I, J), indicating that the WM score was strongly associated with EMT. Due to the important role of EMT in cancer development, a number of studies have explored a series of EMT-related signatures [28–30]; for instance, Wang et al. built an EMT-CYT Index (ECI) model as an indicator for pan-cancer prognosis [30]. We also compared our WM score with EMT score developed by Wang et al. according to hazard ratio, C-index and AUC. The EMT score was calculated for each sample in TCGA-STAD dataset. Although the EMT signature showed a more favorable result in hazard ratio, our WM score had higher C-index and AUC, which supported its robustness (Additional file 8: Fig. S8).

### WM score was related to tumor mutation burden

**Fig. 6:**
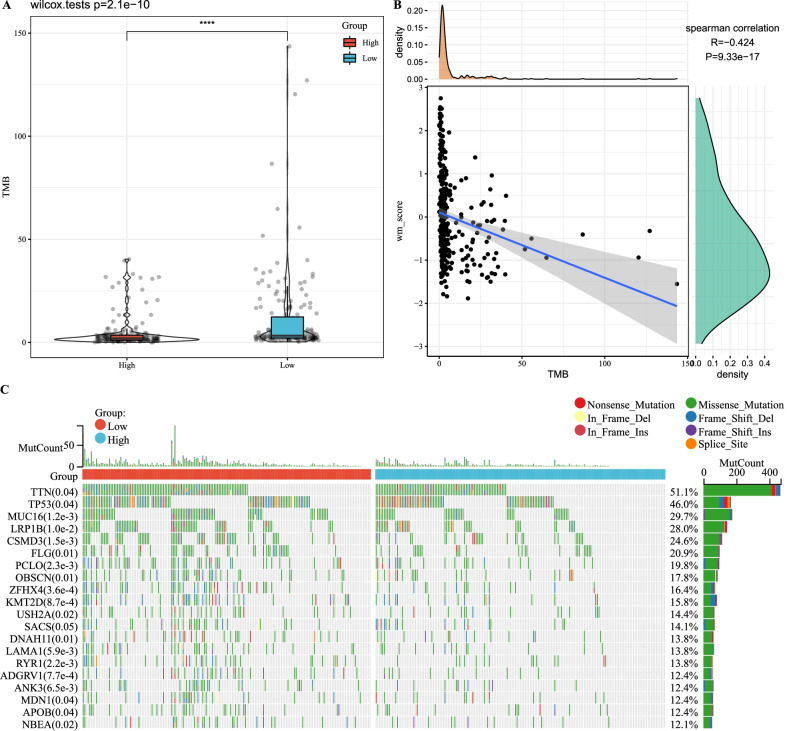
Mutation patterns between high-WM and low-WM score groups. **A** The difference of TMB between high- and low-WM score groups. Wilcoxon test was conducted. **B** Spearman rank correlation of WM score and TMB. R indicates coefficient. **C** The proportion of seven types of mutations (missense, nonsense, in-frame deletions and insertions, frame-shift deletions and insertions, splice site mutation) in high- and low-WM score groups. Fisher’s exact test was conducted. The left column listed the top 20 mutated genes with *p* value in the brackets. The proportion of mutated frequency was presented in the right column. *TMB* tumor mutation burden

Tumor mutation burden (TMB) has been considered to be correlated with tumor immune infiltration, and this can provide a guidance to immunotherapy to some extent. The group with low WM score had higher TMB than that with high WM score in TCGA-STAD dataset (Fig. 6A), and a negative correlation of *R* = − 0.424 (*p* = 9.33e−17, Fig. 6B) between WM score and TMB was observed. The top 20 significantly mutated genes were listed with their *p* value and proportion (Fig. 6C). Missense mutations accounted for the greatest proportion among these mutation types. Frame-shift deletions frequently occurred in *TTN*, *TP53* and *KMT2D*, and nonsense mutations were found the most in *TTN* and *TP53*. Tumor-related genes, such as *TP53*, *LRP1B* and *MUC16,* showed great mutations in both groups but apparently more in low-risk group.

### The relation between WM score and micro-RNA regulation

**Fig. 7:**
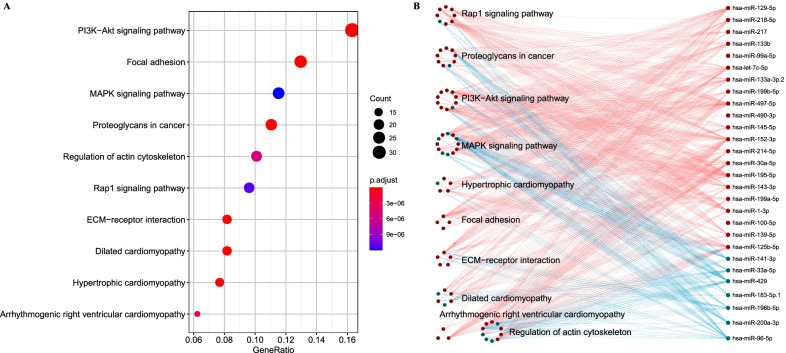
Differential miRNA expression in different RNA modification patterns. **A** The top 10 KEGG pathways enriched from miRNA-targeted genes. The dot size represents the count numbers of genes. **B** Differential expression of miRNA-targeted genes between high- and low-WM score groups. Red and green lines represent low miRNA expression in high-WM score group and high-miRNA expression in low-WM score group. Red and green dots represent highly expressed miRNA-targeted genes in high- and low-WM score groups. The dots within circles mean the miRNA-targeted genes in a pathway

Previous studies demonstrated that 3′-UTR alterations caused by APA will affect posttranscriptional regulation and translation, which could result in loss of miRNA binding sites [31, 32]. The WM score system was developed based on 26 types of RNA modification writers, and we speculated that there was a correlation between WM score and miRNA regulation. Firstly, 46 differentially expressed miRNAs were screened between high-WM score and low-WM score groups. Then, miRNA-targeted genes of these miRNAs were enriched in KEGG pathways using GSVA. In the top 10 enriched pathways, the target genes of miRNAs were highly enriched in PI3K-Akt signaling pathway, focal adhesion and MAPK signaling pathway (Fig. 7A). Moreover, miRNA-targeted genes were differentially expressed between the two groups in these signaling pathways (Fig. 7B), suggesting that WM score was related to the regulation of miRNA-related signaling pathways.

### The sensitivity of chemotherapeutic drugs was related to WM score

**Fig. 8:**
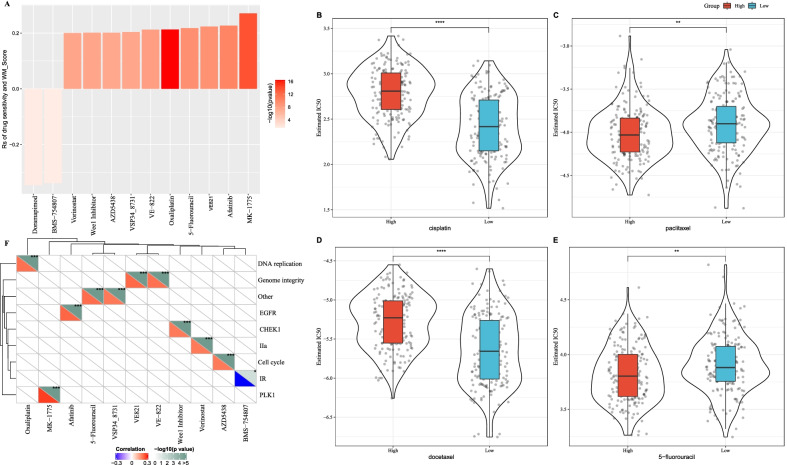
The applicative value of WM score in chemotherapy. **A** Screening of chemotherapeutic drugs showing a correlation with WM score. |rs|  > 0.2 was considered to have a correlation, where rs represents Spearman rank correlation coefficient. **B**–**E** The difference of drug sensitivity of cisplatin (**B**), paclitaxel (**C**), docetaxel (**D**) and 5-flurouracil between high-WM score and low-WM score groups. **F** The targeted pathways of chemotherapeutic drugs showing a correlation with WM score. Student’s *t* test was performed. **p < 0.01, ****p < 0.0001

To examine whether WM score and patients’ sensitivity of chemotherapeutic drugs was correlated, we exported a series of drugs for treating GC from GDSC database. Spearman rank correlation analysis identified 12 chemotherapeutic drugs significantly correlated with WM score. Among these drugs, WM score showed close relations to the sensitivity of two drugs (p38 MAPK inhibitor doramapimod and IGF-1R inhibitor BMS-754807) and to the resistance of 10 drugs (Fig. 8A). Furthermore, pRRophetic R package was used to evaluate the drug response of TCGA-STAD samples to cisplatin, paclitaxel, docetaxel and 5-fluorouracil. Low score of estimated IC50 represented a high sensitivity to drugs. Low-WM score group showed high sensitivity to cisplatin and docetaxel, and patients with a high-WM score were more sensitive to paclitaxel and 5-fluorouracil (Fig. 8B–E). The targeted pathways of chemotherapeutic drugs were also investigated. Here, each drug targeted different pathways, such as IGF-1R inhibitor, and BMS-754807 and oxaliplatin were related to IR signaling pathway and DNA replication pathway, respectively (Fig. 8F).

### The efficacy of anti-PD-1 therapy is associated with WM score

**Fig. 9:**
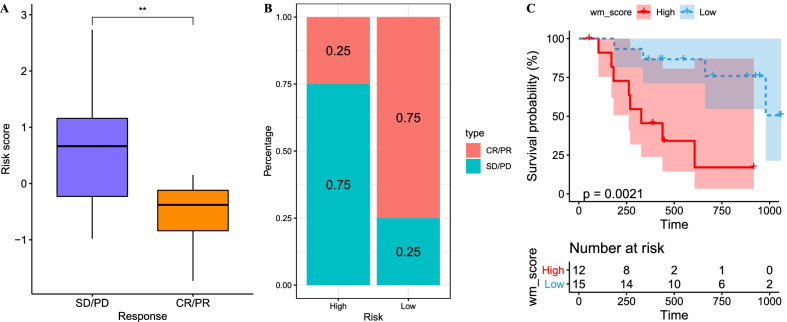
The prognostic value of WM score for immunotherapy. **A** The difference of WM score between CR/PR samples and SD/PD samples in GSE78220 dataset. Student’s *t* test was performed. **B** The proportion of CR/PR samples and SD/PD samples in high- and low-WM score groups in GSE78220 dataset. **C** Survival analysis of high- and low-WM score groups in GSE78220 dataset. Log-rank test was performed in survival analysis. **p < 0.01

For metastatic cancer, immunotherapy, especially anti-PD-1/PD-L1 therapy, is a promising strategy in treating many cancer types. We therefore examined the WM score in predicting the efficacy of immunotherapy. In GSE78220 dataset, 27 patients with four outcome information of complete response (CR), partial response (PR), stable disease (SD) and progressive disease (PD) treated by anti-PD-1 therapy were included. The result presented that CR and PR patients had a lower WM score than SD and PD patients (*p* < 0.01, Fig. 9A), and a higher proportion of CR and PR patients was in low-WM score group (Fig. 9B). Kaplan–Meier survival plot also displayed a favorable OS in low-WM score group (*p* = 0.021, Fig. 9C), suggesting that WM score was closely associated with the outcomes of immunotherapy.

## Discussion

Writers, readers and erasers are the three different types of RNA modifications, and their effect on stimulating or suppressing tumor growth is regarded as hallmarks of cancers [19]. Studies have proposed various signatures of RNA modification writers such as METTL3 [33], NSUN2 [34] and a signature of 3 m^6^A regulators (FTO, RBM15, ALKBH5) [8] to predict GC prognosis. This study focused on a series of writers including m^1^A, m^6^A, APA and A-to-I writers capable of modifying adenosine. A total of 26 writers were included in the present study, and the assessment of multiple aspects on the writers confirmed their clinical significance in GC survival and treatment.

Compared with normal samples, high expression of 24 writers in GC samples was detected (Fig. 1E). Mutant group and WT group showed differential OS (Fig. 1B), suggesting that these mutations on RNA writers possibly had an important effect on their expression level or function, which also contributed to tumor development. Exploration on the GEO database showed that many writers differentially expressed in gastric cancer were also differentially expressed in other cancer types. For example, METTL3 is significantly up-regulated in esophageal squamous cell carcinoma, and this leads to a worse prognosis through activating Notch signaling pathway [35]. Wang et al. discovered that the inhibition of METTL3 or METTL14 can help colorectal cancer and melanoma cell acquire an enhanced immune response to anti-PD-1 therapy through increasing CD8 T cells and IFN-γ secretion [36]. Such an observation revealed that METTL3 or METTL14 could serve as potential targets for assisting immune checkpoint blockade therapy. WTAP is a nuclear protein that facilitates cell proliferation and apoptosis. WTAP expression is elevated in cholangiocarcinoma, and WTAP knockdown significantly suppresses tumorigenesis in a xenograft model [37]. KIAA1429, the largest known component in the m6A methyltransferase complex, is overexpressed in hepatocellular carcinoma tissues and is associated with poor prognosis [38], which is consistent in GC.

Based on the discovery that different expression patterns of RNA modification writers were related to different OS, we constructed a WM scoring system with high-performance prognosis prediction. Previous researches have illustrated that RNA modifications can regulate immune functions such as immune recognition and immune responses [39, 40]. TME is a decisive factor of tumor development and could affect the efficacy of targeted treatment such as immunotherapies. According to the infiltration of cytotoxic lymphocytes (CTLs) and tumor-associated macrophages (TAMs) in tumor cells, TME can be divided into immune-inflammation, immune-exclusion and immune-desert [41]. Immune-inflammation type shows abundant expressions of PD-L1 and activated CTLs; therefore, patients of this type can benefit the most from the anti PD-1/PD-L1 therapy [42]. This study presented that high-WM score group had a higher enrichment score of immune cells, indicating that patients in high-WM score group could benefit more from the anti-PD-1/PD-L1 blockade. Moreover, as high- and low-WM groups demonstrated significantly different OS after receiving anti-PD-1/PD-L1 therapy, the WM scoring system may have a potential in predicting patients who may develop favorable outcome from the targeted therapy. Although no treatment data of GC patients with anti-PD-1/PD-L1 therapy could be obtained, the results still provided a possibility that WM score can guide immunotherapy.

EMT plays an essential role in embryonic development and allows epithelial cells to transform into mesenchymal cells [43]. EMT also participates in tumor progression, metastasis, recurrence and chemoresistance [44]. EMT process varies greatly in different cancer types, which is possibly related to different EMT-related pathways activated by cancers. Notably, cytokines, chemokines and growth factors secreted by stromal cells and immune cells can induce EMT process, showing a close relation between tumor microenvironment and EMT [44, 45]. A number of studies developed various signatures related to EMT in pan-cancer or different cancer types [29, 46, 47]. For instance, Dai et al. established an EMT-related signature for predicting GC prognosis, and five EMT-related prognostic genes were found to be highly expressed in GC tissues [48]. Wang et al. proposed an EMT-CYT Index (ECI) model capable of predicting prognosis for pan-cancer patients [30]. Our WM score manifested a better performance in prognosis prediction when compared with the EMT signature of Wang et al. Notably, EMT has a correlation with ECM remodeling [49], and this also supports our result that ECM–receptor interaction pathway was highly enriched in differentially expressed miRNAs between high-WM and low-WM score groups.

Although various EMT-related signatures were developed for many cancer types in previous studies, our WM scoring system further validated the importance of EMT in cancer progression and discovered the cross talk between RNA modification and EMT. Consistent with the previous findings, in this study, EMT pathways were highly activated in the high-WM score group, which also had a high immune enrichment score. The result indirectly proved the close relation between TME and EMT. In accordance with the previous observation that EMT process can promote tumor metastasis, the current research confirmed the relation between worse prognosis and highly activated EMT-related pathways in the high-WM score group.

RNA modification writers play a critical role in regulating biological process in cancer. Writers such as METTL3 and METTL14 show cancer-promoting or cancer-suppressive effects on the same or different cancer types [50–53]. To some extent, this may interfere the development of WM scoring system. Another limitation of the study was that the anti-PD-1/PD-L1 treatment data came from melanoma, as no available anti-PD-1/PD-L1 treatment data can be obtained from GC.

Nevertheless, this study still expanded the research on the effect of adenosine-related RNA modifications on GC development and pathogenesis. These RNA modifications were closely involved in altering TME and TME through oncogenic pathways such as proteoglycans, PI3K-Akt signaling, MAPK signaling, and ECM-receptor interaction pathways. High-WM and low-WM score groups manifested significant differences in GC patients’ sensitivity to chemotherapy and immunotherapy. Hence, the WM scoring system in this study showed a great potential in predicting GC prognosis and could assist the decision-making of targeted therapies.

## Conclusion

In conclusion, this study constructed a WM scoring system based on four types of RNA modification writers. The WM scoring system exhibited robust performance in all the datasets explored. Overall, high-WM and low-WM score groups classified by the WM scoring system manifested distinct prognostic difference, immune infiltration, EMT-related pathways, gene mutation and sensitivity to chemotherapy and immunotherapy. We concluded that adenosine-related RNA modifications were closely associated with tumorigenesis and pathology in GC possibly through modulating tumor microenvironment and facilitating EMT. Notably, the WM scoring system had the ability to assist and guide chemotherapy and immunotherapy, especially for metastatic GC patients.

## Methods

### Data information

GC samples were exported from Gene Expression Omnibus (GEO) database (https://www.ncbi.nlm.nih.gov/geo/) and The Cancer Genome Atlas database (TCGA, https://portal.gdc.cancer.gov/). GSE62254 dataset containing gene expression data and clinical information were obtained from GEO (Additional file 9: Table S1). TCGA-STAD dataset with mRNA expression, miRNA expression, somatic cell mutations, somatic copy number amplifications (SCNAs) and clinical features including stages, sex, age and survival data was downloaded from TCGA (Additional file 10: Table S2). GSE78220 dataset acquired from GEO contained the treatment data of anti-PD-1 therapy for melanoma.

### Unsupervised consensus clustering

Unsupervised consensus clustering is a widely used method for providing quantitative and visual stability evidence for estimating the number of unsupervised classes in a dataset [54]. We used ConsensusClusterPlus R package to conduct unsupervised consensus clustering on 26 types of writers in GSE62254 dataset [54]. Thousand repeats were performed to produce stable clusters. Writers included 7 m^6^A enzymes (KIAA1429, METTL3, METTL14, RBM15, RBM15B, WTAP and ZC3H13), 4 m^1^A enzymes (TRMT6, TRMT10C, TRMT61A and TRMT61B), 12 APA enzymes (CFI, CLP1, CPSF1-4, CSTF1/2/3, NUDT21, PABPN1 and PCF11) and 3 A-to-I enzymes (ADAR, ADARB1 and ADARB2).

### Functional analysis on clusters 1 and 2

Gene set variation analysis (GSVA) was performed to assess the enrichment of mutant and wild-type RNA modification genes in “h.all.v7.2” and the enriched KEGG pathways of clusters 1 and 2 in “c2.cp.kegg.v7.1” using GSVA R package [55]. PathScore [56] was employed to evaluate the enrichment of molecular function and biological process of RNA modification genes in “c5.bp.v7.1”, “c5.mf.v7.1.” Reference gene sets of “c2.cp.kegg.v7.1,” “c5.bp.v7.1,” “c5.mf.v7.1” and “h.all.v7.2” were downloaded from MSigDB database (https://www.gsea-msigdb.org/gsea/msigdb). ClusterProfiler R package was used to annotate gene ontology terms on differentially expressed genes between clusters 1 and 2 [57]. Benjamini and Hochberg (BH) method was used to correct false discovery rate (FDR) in function annotations, with FDR < 0.05 as a significance.

### Immunohistochemistry (IHC) for evaluating RNA writer expression in GC tissues

IHC could identify cellular or subcellular distribution and expression of one objective protein based on monoclonal or polyclonal antibodies. This study performed IHC to verify the expression of ADARB1 (Affbiotech, Catalog number: DF3227, dilution: 1:100) and RBM15 (Proteintech, Catalog number: 10587-1-AP, dilution: 1:200) in early and advanced GC tissues following the general procedures. Twenty pairs of GC and paired normal tissue samples were obtained from The Second Affiliated Hospital of Guangzhou Medical University. Written informed consent was obtained from all patients. The experimental procedures were approved by the Institutional Ethics Committee of The Second Affiliated Hospital of Guangzhou Medical University.

### Analysis of immune infiltration

CIBERSORT (http://cibersort.stanford.edu/) was used to analyze the distribution and enrichment score of the 22 immune cells [58]. The CIBERSORT tool could estimate the fraction of 22 immune cells in complex tissues according to gene expression profiles, and it has been applied in a number of studies for characterizing tumor microenvironment in cancers.

### Construction of a WM scoring system

Firstly, differentially expressed genes (DEGs) between cluster 1 and cluster 2 in GSE62254 dataset were identified using Limma R package [59]. The expressions of 194 DEGs were used as input to conduct unsupervised consensus clustering in ConsensusClusterPlus R package [54]. Cluster number k = 2 was selected to construct a consensus matrix. A WM scoring system based on Writers of RNA Modifications was presented as WM score = *Σ*(coefficient *i*) * (expression *i*); here, *i* represented differentially expressed genes significantly related to prognosis [27]. High- and low-WM score groups were classified by the median of WM score. The effectiveness of the WM scoring system was validated by ROC through timeROC package and multivariate Cox regression analysis in survival R package [60]. TCGA-STAD was used as a validation dataset to assess the robustness of the WM scoring system.

### Gene mutation analysis

To characterize the difference of gene mutations between high-WM and low-WM groups, mutect2 software (https://software.broadinstitute.org/cancer/cga/mutect) was employed to analyze the gene mutations in TCGA-STAD dataset. Mutect2 could preprocess high-throughput sequencing data and detect mutations with a very low false positive rate. CancereffectsizeR package [61] was used to calculate mutation frequency, and the top 20 significantly mutated genes were screened by Fisher’s exact test.

### Spearman rank correlation analysis

Spearman rank correlation analysis was applied to analyze the correlation among 26 writers, WM score and the sensitivity to chemotherapeutic drugs. The data of drug sensitivity were downloaded from Genomics of Drug Sensitivity in Cancer (GDSC, https://www.cancerrxgene.org/) database [62]. |rs| > 0.2 was considered to show a correlation, where rs = Spearman rank correlation coefficient. FDR was corrected by BH correction (FDR < 0.05 was significant). Drug response was evaluated by pRRophetic R package [63].

### Analysis of miRNA expression, miRNA-targeted genes and pathways

The miRNA expression data were exported from TCGA-STAD dataset. Differential expressed miRNAs between high- and low- WM score groups were screened by Limma R package. Then, GSVA R package was applied to analyze miRNA-targeted genes and KEGG functional pathways in “c2.cp.kegg.v7.1.” Wilcoxon test was performed to assess differences between the two groups. FDR was corrected by BH correction, and FDR < 0.05 was considered as a significance.

### Statistical analysis

All the statistical analyses were performed in the R (3.6.2). Parameters of measurements were default if there was no special description. Statistical methods were shown in the corresponding figure legends. *p* < 0.05 was considered to be significant.

## Supplementary Information

**Additional file 1: Fig. S1.** Enriched pathways of significantly mutated genes evaluated by PathScore.

**Additional file 2: Fig. S2.** IHC results of ADARB1 and RBM15B in gastric cancer tissues. (A-B) IHC of ADARB1 in early gastric cancer tissue. (C–D) IHC of ADARB1 in advanced gastric cancer tissue. (E–F) IHC of RBM15B in early gastric cancer tissue. (G–H) IHC of RBM15B in advanced gastric cancer tissue. (I–J) Sections were semi-quantitatively scored for ADARB1 or RBM15B staining patterns as follows: the staining extent in each core was scored as 1+ (< 25% staining of tumor cells), 2+ (25–50% staining of tumor cells), 3+ (50% to 75% staining of tumor cells), or 4+ (> 75% staining of tumor cells). Additionally, the staining intensity was quantified as 0 (negative), 1+ (weak), 2+ (intermediate), or 3+ (strong). The final immunoreaction score was obtained by multiplying the intensity and extension values (range 0–12) and the samples were grouped as 1+ (score 0–2), 2+ (score 3–4), 3+ (score 6–8) and 4+ (score 9–12) staining. Meanwhile, for statistical purposes, scores of 3+ and 4+ was defined as high expression and the other final scores were considered as low expression, and then chi-squared test was used to compare the differences between high-expression and low-expression groups.

**Additional file 3: Fig. S3.** The top 10 significantly enriched BP terms (A) and MF (B) terms of clusters 1 and 2. FDR < 0.05.

**Additional file 4: Fig. S4.** The correlation between 26 RNA modification writers and immune infiltration. *p < 0.05, **p < 0.01, ***p < 0.001, ****p < 0.0001.

**Additional file 5: Fig. S5.** Evaluation of immune infiltration of two clusters by Timer. ***p < 0.001.

**Additional file 6: Fig. S6.** Functional analysis of 194 RNA modification related genes and survival analysis of 300 samples in GSE62254 dataset. (A-C) GO analysis of 194 genes on molecular function (A), cellular component (B) and biological process (C). (D) Kaplan-Meier survival curve of cluster A and cluster B in GSE62254 dataset. Log-rank test was performed.

**Additional file 7: Fig. S7.** Comparison of WM score and EMT score on hazard ratio, C-index, and 1-year, 3-year and 5-year AUC. CI, confidence interval. AUC, area under ROC curve.

**Additional file 8: Fig. S8.** Comparison of WM score and EMT score on hazard ratio, C-index, and 1-year, 3-year and 5-year AUC. CI, confidence interval. AUC, area under ROC curve.

**Additional file 9: Table S1.** Clinical information of TCGA-STAD dataset.

**Additional file 10: Table S2.** Clinical information of GSE62254 dataset.

## Data Availability

The datasets generated during and/or analyzed during the current study are available in the [GSE62254] repository [https://www.ncbi.nlm.nih.gov/geo/query/acc.cgi?acc=GSE62254] and in [GSE78220] repository [https://www.ncbi.nlm.nih.gov/geo/query/acc.cgi].

## Abbreviations

AML: Acute myeloid leukemia
APA: Alternative polyadenylation
ASR: An age-standardized incidence rate
A-to-I: Adenosine-to-inosine
AUC: Area under ROC curve
BH: Benjamini and Hochberg
CNV: Copy number variation
CR: Complete response
CTLs: Cytotoxic lymphocytes
DEGs: Differentially expressed genes
DMS: A DNA methylation score
EMT: Epithelial–mesenchymal transition
FDR: False discovery rate
GC: Gastric cancer
GDSC: Genomics of Drug Sensitivity in Cancer
GEO: Gene Expression Omnibus
GO: Gene Ontology
GSVA: Get set variation analysis
IC50: The biochemical half maximal inhibitory concentration
IHC: Immunohistochemistry
KEGG: Kyoto Encyclopedia of Genes and Genomes
m^1^A: N1-methyladenosine
m^6^A: N6-methyladenosine
MSigDB: The Molecular Signatures Database
OS: Overall survival
PD: Progressive disease
PD-1: Programmed cell death protein 1
PD-L1: Programmed cell death ligand 1
PR: Partial response
ROC: Receiver operating characteristic curve
rRNA: Ribosomal RNA
SCNAs: Somatic copy number amplifications
SD: Stable disease
TAMs: Tumor-associated macrophages
TCGA: The Cancer Genome Atlas
TMB: Tumor mutation burden
TME: Tumor microenvironment
WM: Writers of RNA Modifications
