# Supplementary figures and images for: Cross talk between RNA modification writers and tumor development as a basis for guiding personalized therapy of gastric cancer
Source: Hum Genomics. 2022 Apr 22;16:14. doi: 10.1186/s40246-022-00386-z (PMC9027049; doi:10.1186/s40246-022-00386-z)

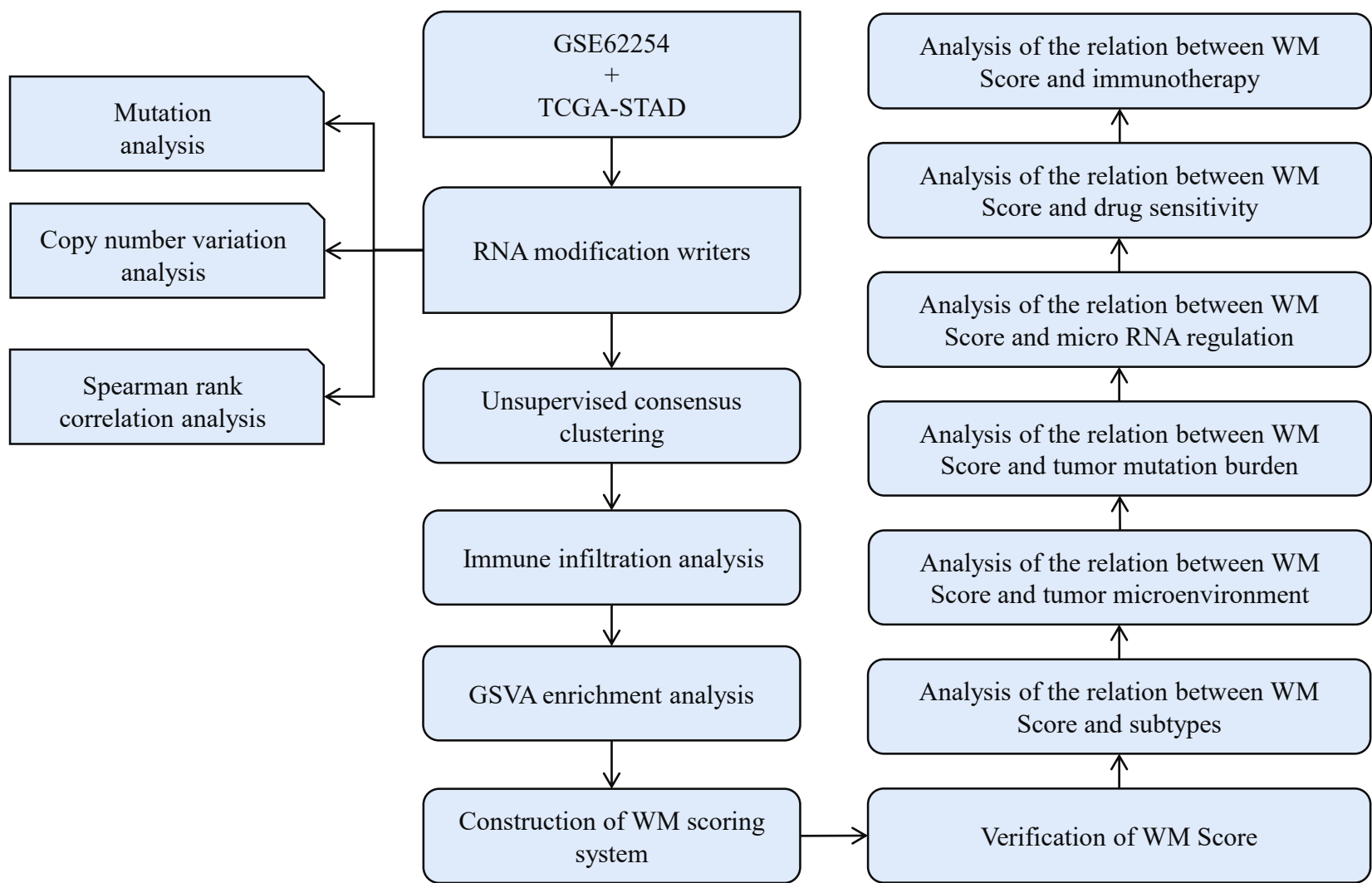

Supplement: Supplementary file 1 — Additional file 1: Fig. S1. Enriched pathways of significantly mutated genes evaluated by PathScore. [file 40246_2022_386_MOESM1_ESM.pdf]

BARD1 PATHWAY  
(56.5% patients, P<1e-16, Q<1e-13)

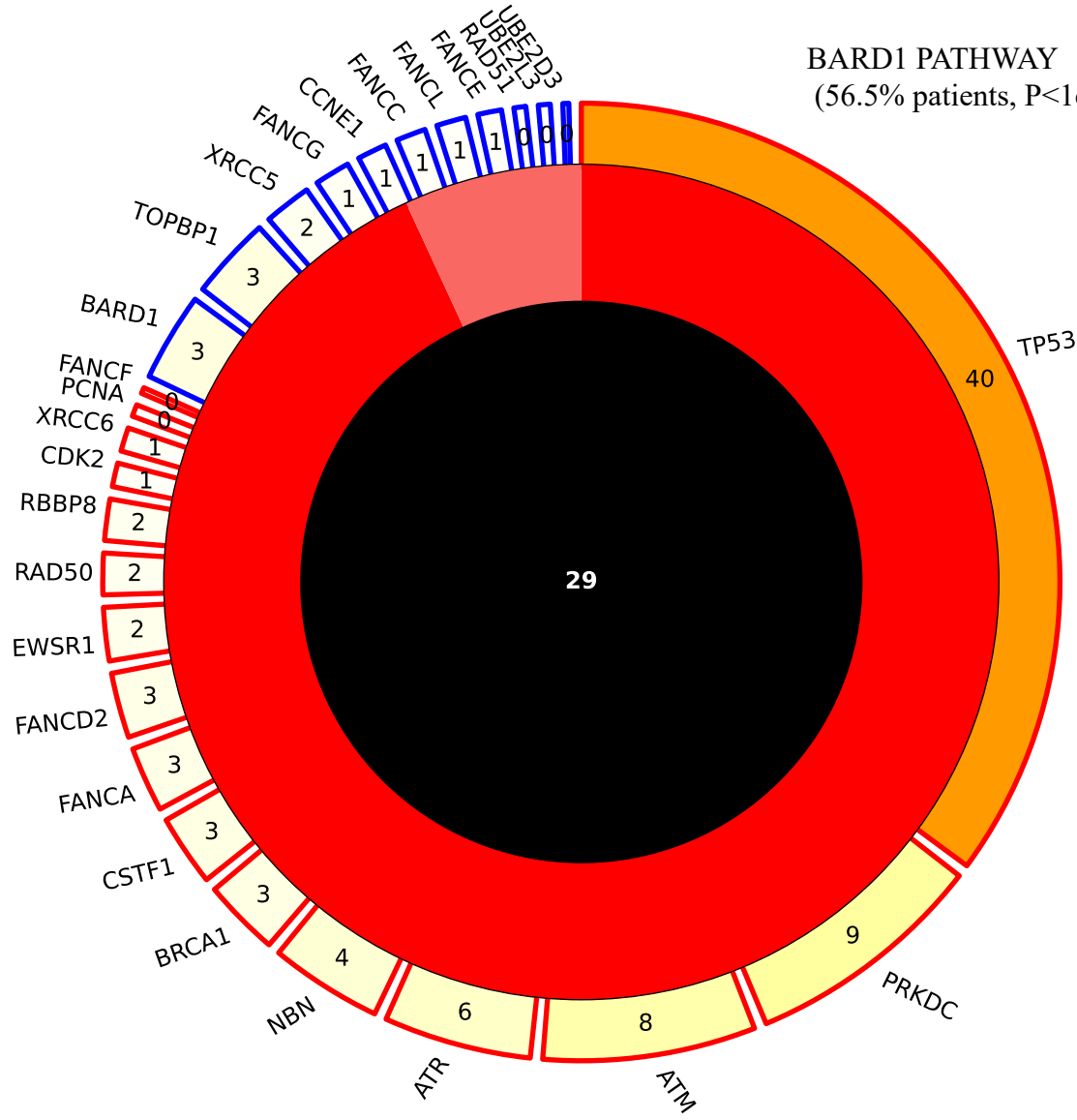

INTERFERON ALPHA BETA SIGNALING  
(33% patients, P=0.048, P\*=1)

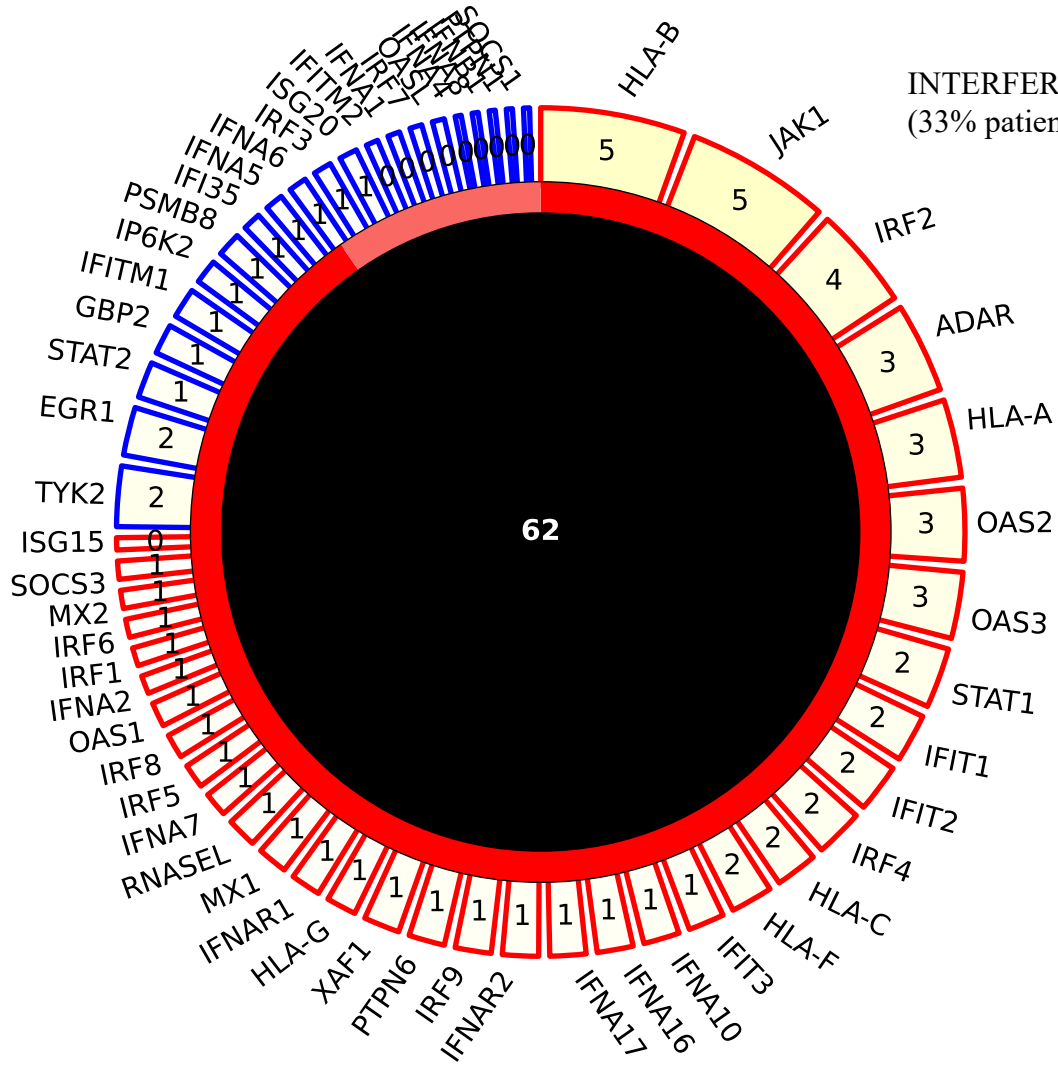

Supplement: Supplementary file 2 — Additional file 2: Fig. S2. IHC results of ADARB1 and RBM15B in gastric cancer tissues. (A-B) IHC of ADARB1 in early gastric cancer tissue. (C–D) IHC of ADARB1 in advanced gastric cancer tissue. (E–F) IHC of RBM15B in early gastric cancer tissue. (G–H) IHC of RBM15B in advanced gastric cancer tissue. (I–J) Sections were semi-quantitatively scored for ADARB1 or RBM15B staining patterns as follows: the staining extent in each core was scored as 1+ (< 25% staining of tumor cells), 2+ (25–50% staining of tumor cells), 3+ (50% to 75% staining of tumor cells), or 4+ (> 75% staining of tumor cells). Additionally, the staining intensity was quantified as 0 (negative), 1+ (weak), 2+ (intermediate), or 3+ (strong). The final immunoreaction score was obtained by multiplying the intensity and extension values (range 0–12) and the samples were grouped as 1+ (score 0–2), 2+ (score 3–4), 3+ (score 6–8) and 4+ (score 9–12) staining. Meanwhile, for statistical purposes, scores of 3+ and 4+ was defined as high expression and the other final scores were considered as low expression, and then chi-squared test was used to compare the differences between high-expression and low-expression groups. [file 40246_2022_386_MOESM2_ESM.pdf]

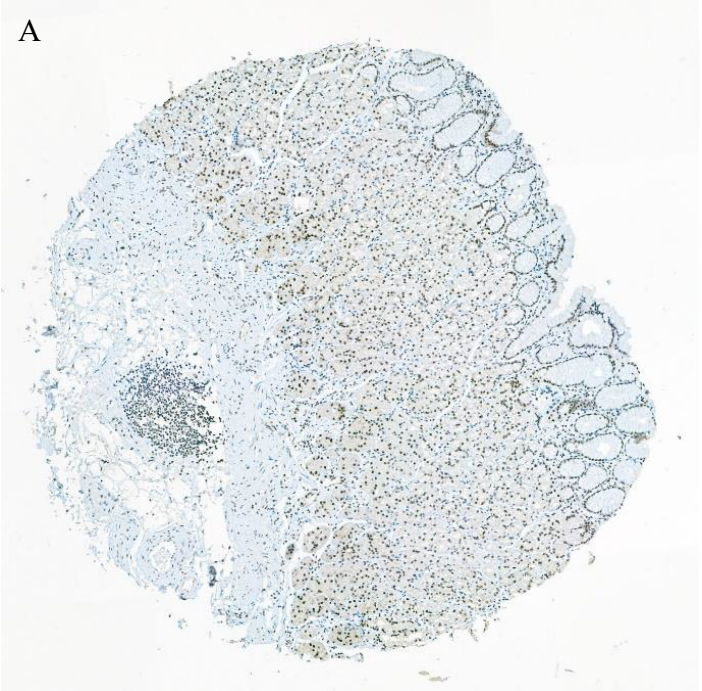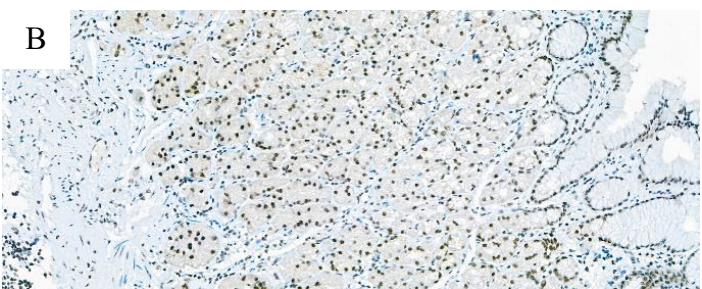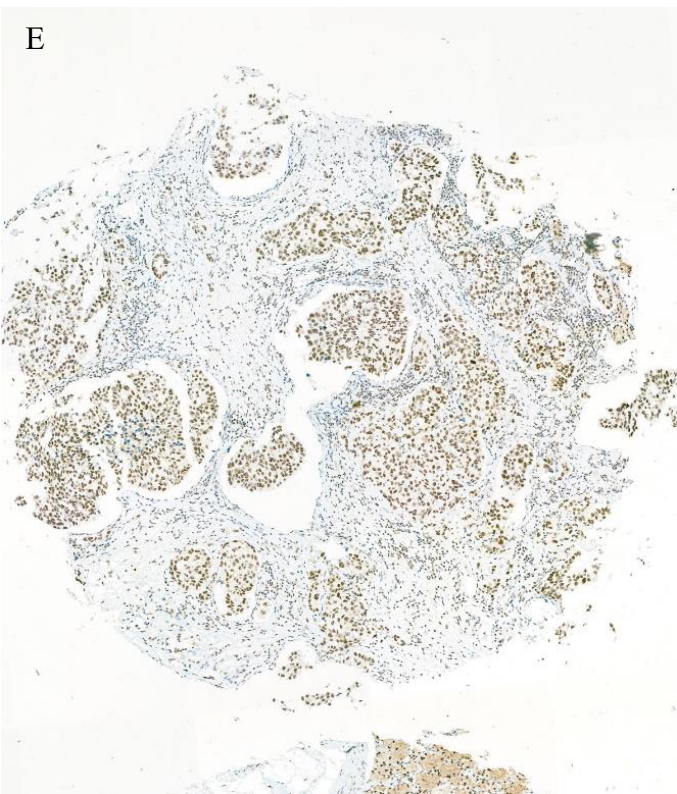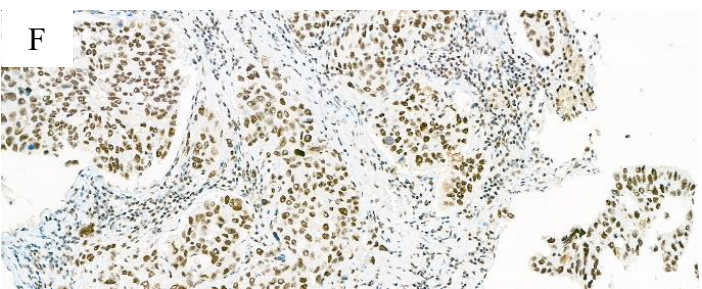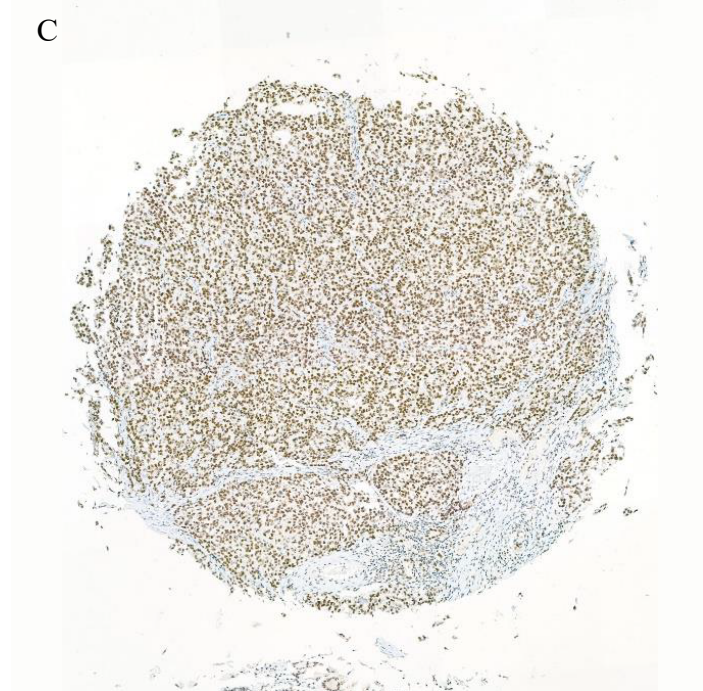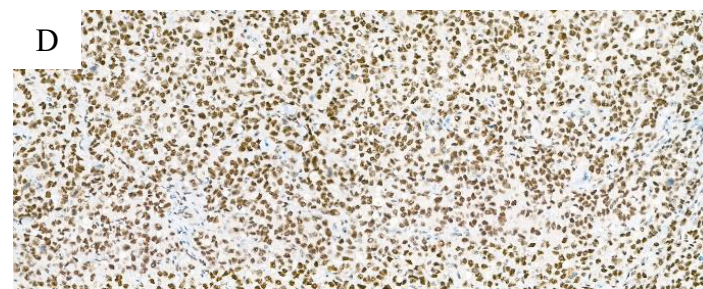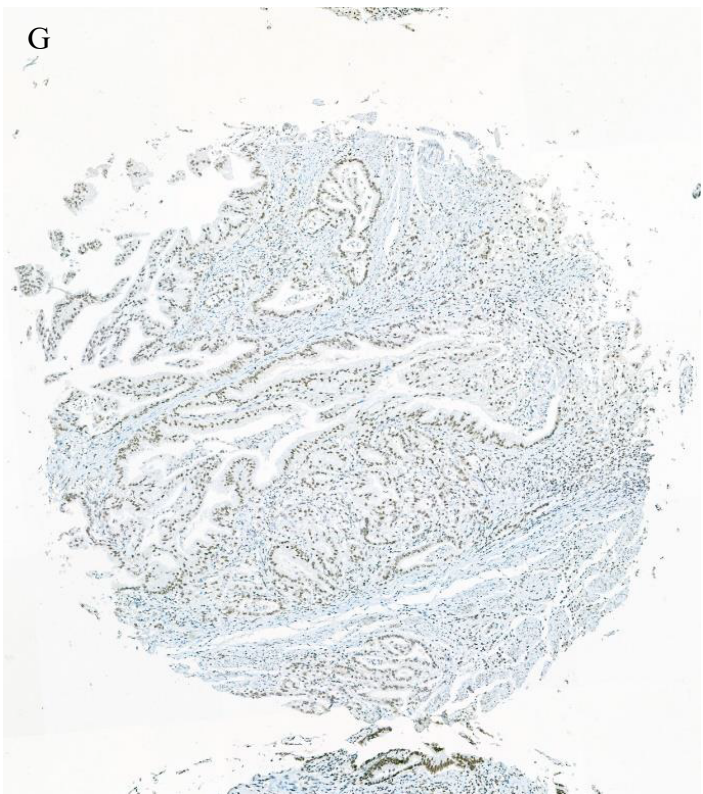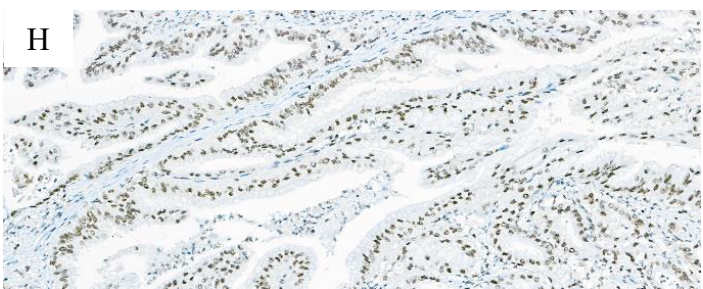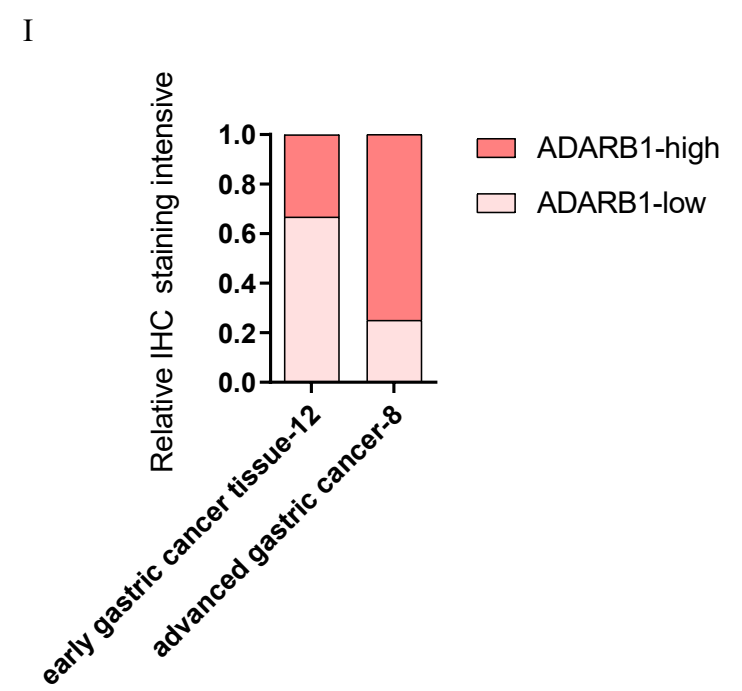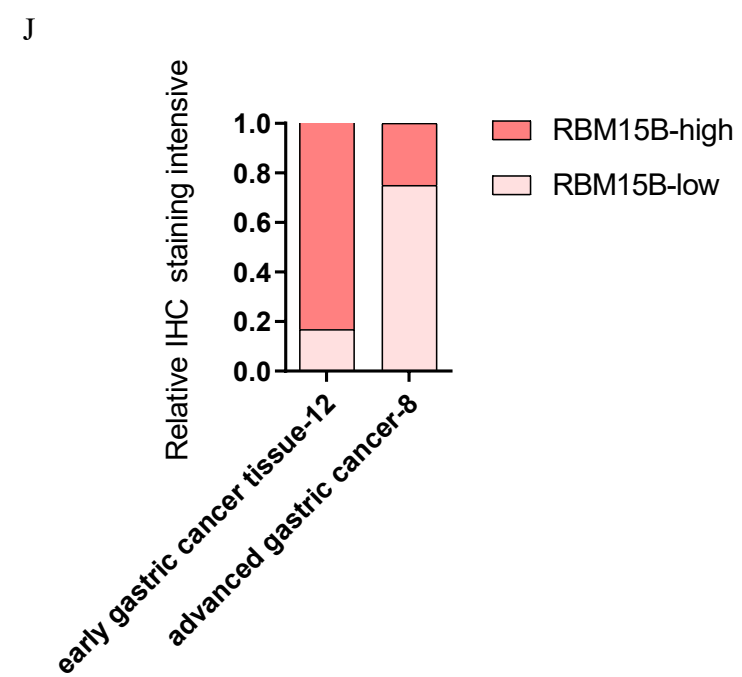

Supplement: Supplementary file 3 — Additional file 3: Fig. S3. The top 10 significantly enriched BP terms (A) and MF (B) terms of clusters 1 and 2. FDR < 0.05. [file 40246_2022_386_MOESM3_ESM.pdf]

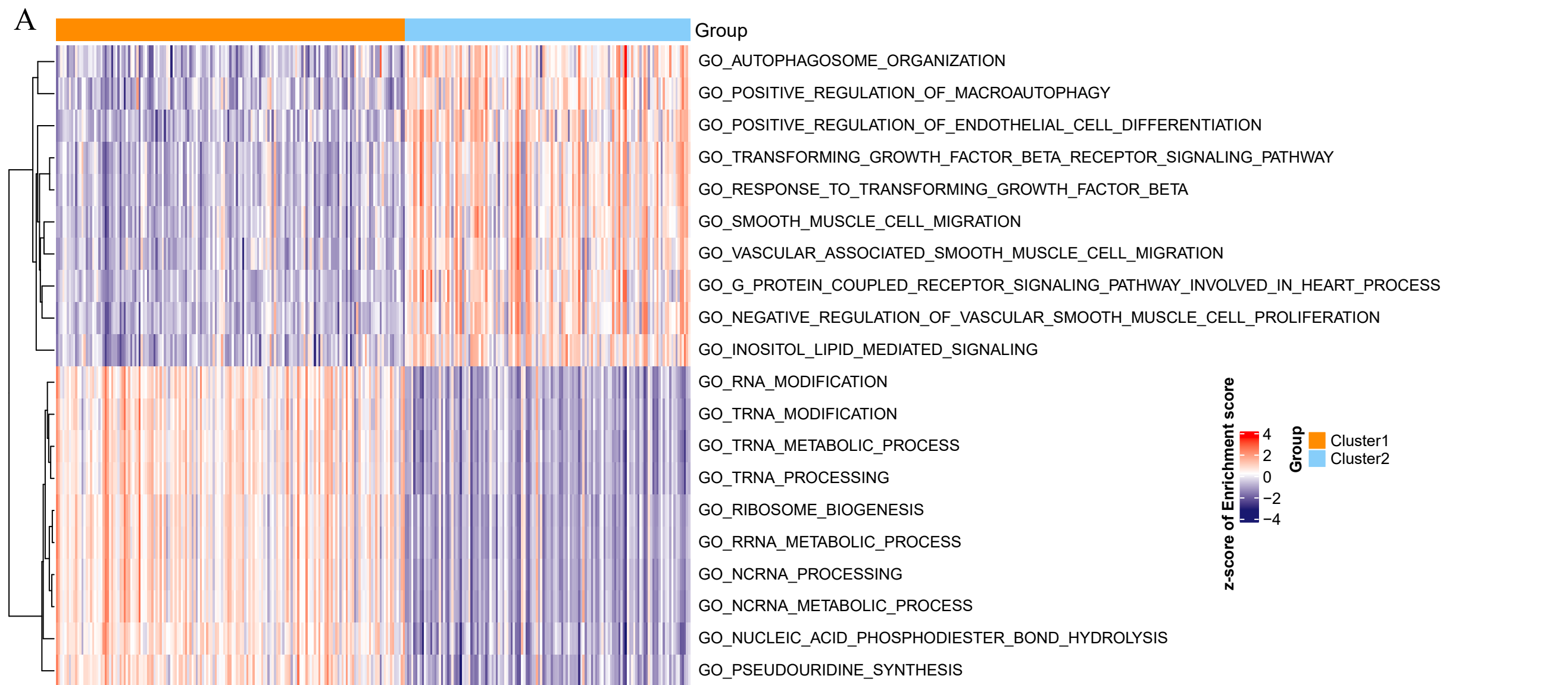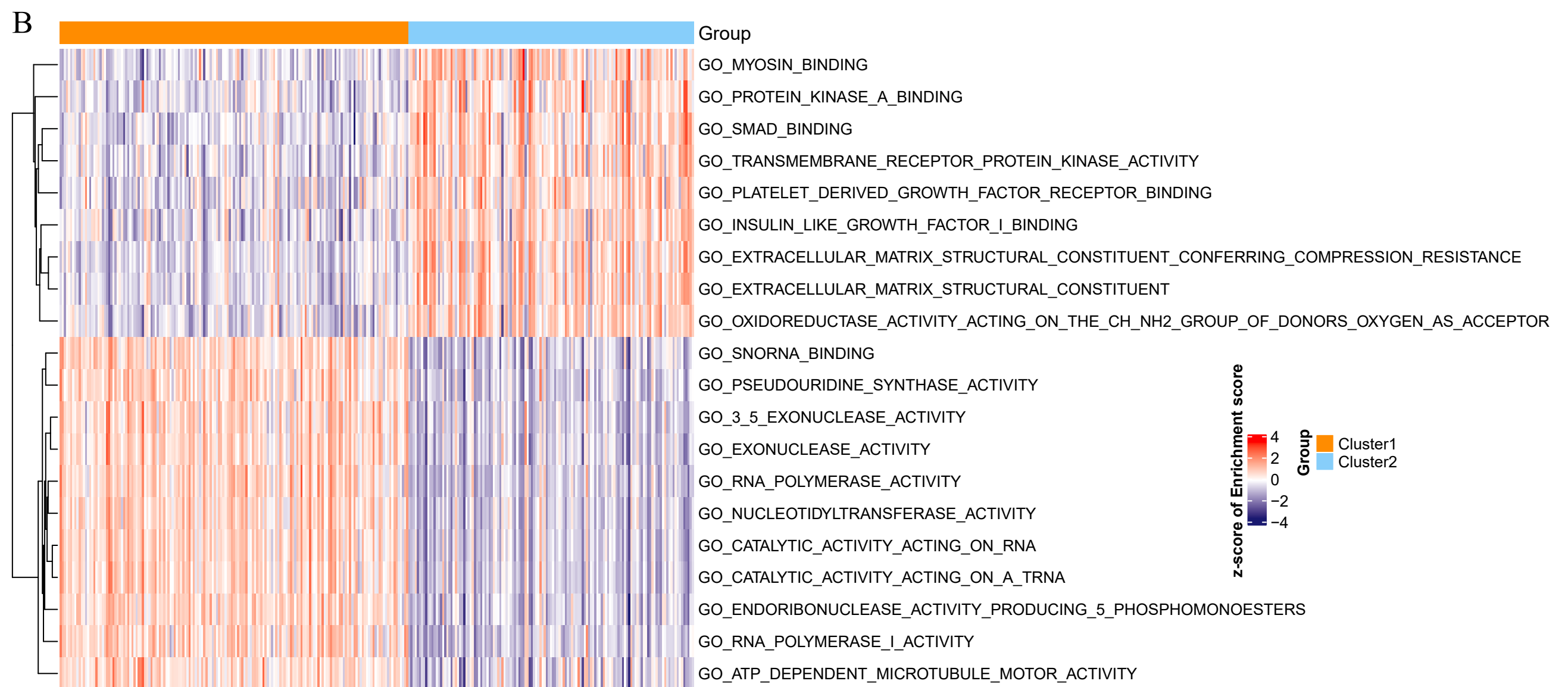

Supplement: Supplementary file 4 — Additional file 4: Fig. S4. The correlation between 26 RNA modification writers and immune infiltration. *p < 0.05, **p < 0.01, ***p < 0.001, ****p < 0.0001. [file 40246_2022_386_MOESM4_ESM.pdf]

Correlation with immune cell type

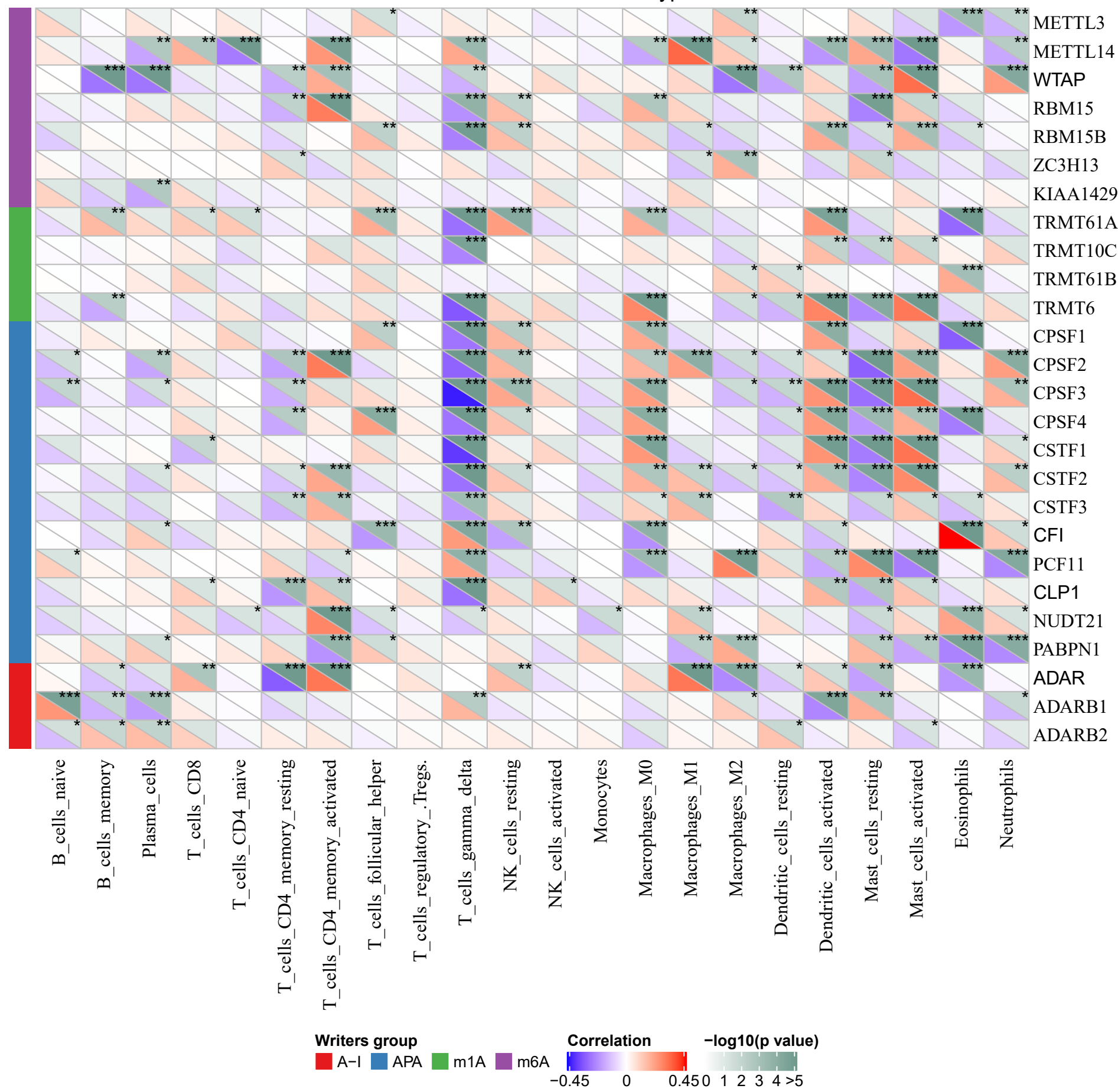

Supplement: Supplementary file 5 — Additional file 5: Fig. S5. Evaluation of immune infiltration of two clusters by Timer. ***p < 0.001. [file 40246_2022_386_MOESM5_ESM.pdf]

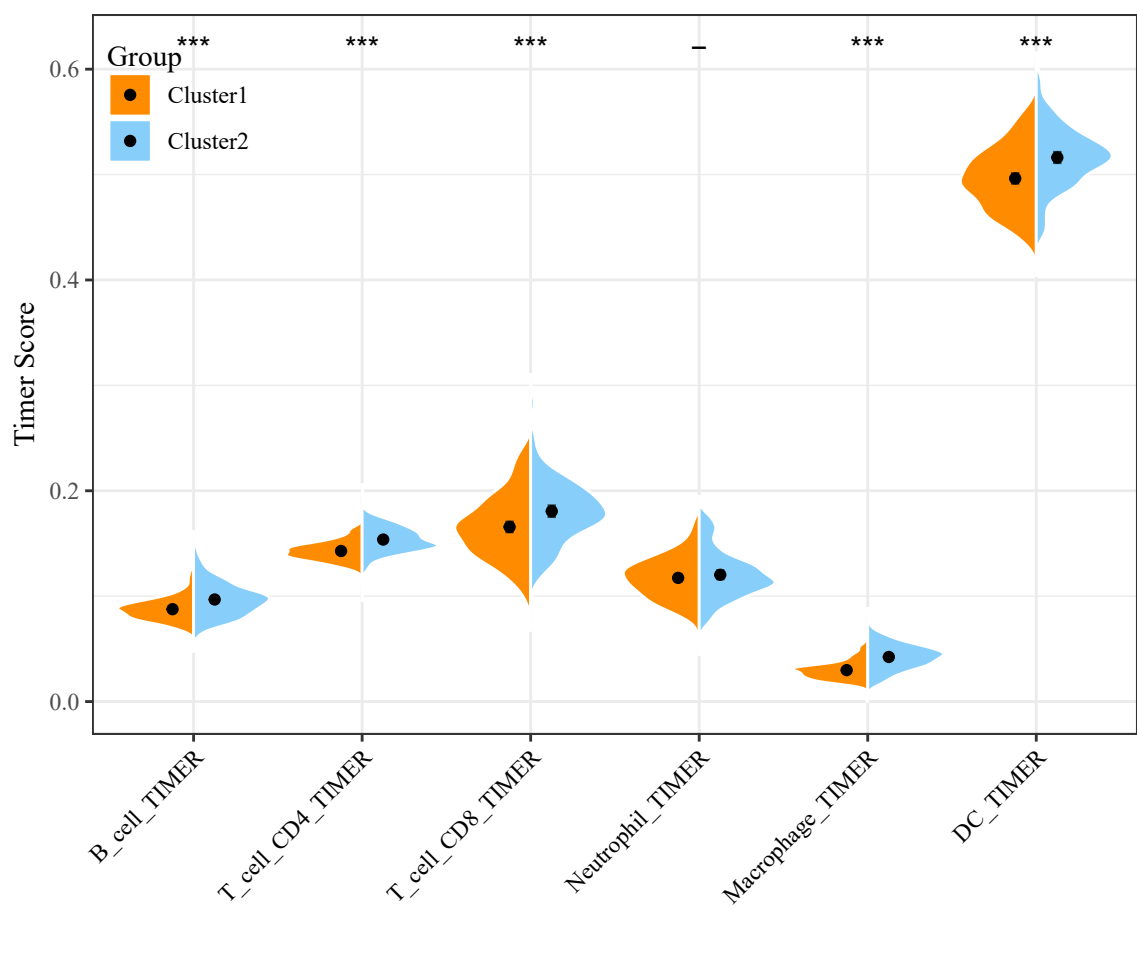

Supplement: Supplementary file 6 — Additional file 6: Fig. S6. Functional analysis of 194 RNA modification related genes and survival analysis of 300 samples in GSE62254 dataset. (A-C) GO analysis of 194 genes on molecular function (A), cellular component (B) and biological process (C). (D) Kaplan-Meier survival curve of cluster A and cluster B in GSE62254 dataset. Log-rank test was performed. [file 40246_2022_386_MOESM6_ESM.pdf]

A

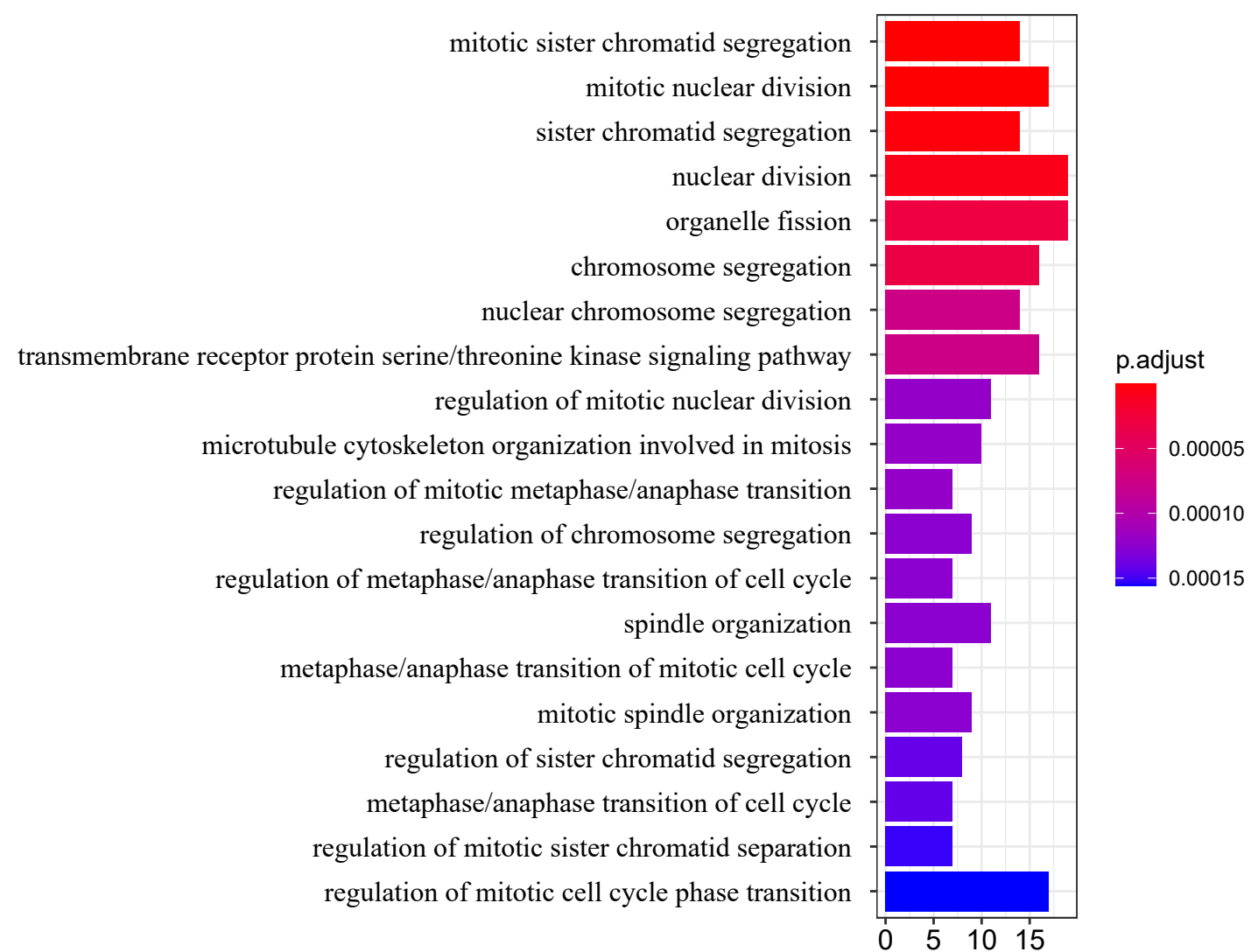

B

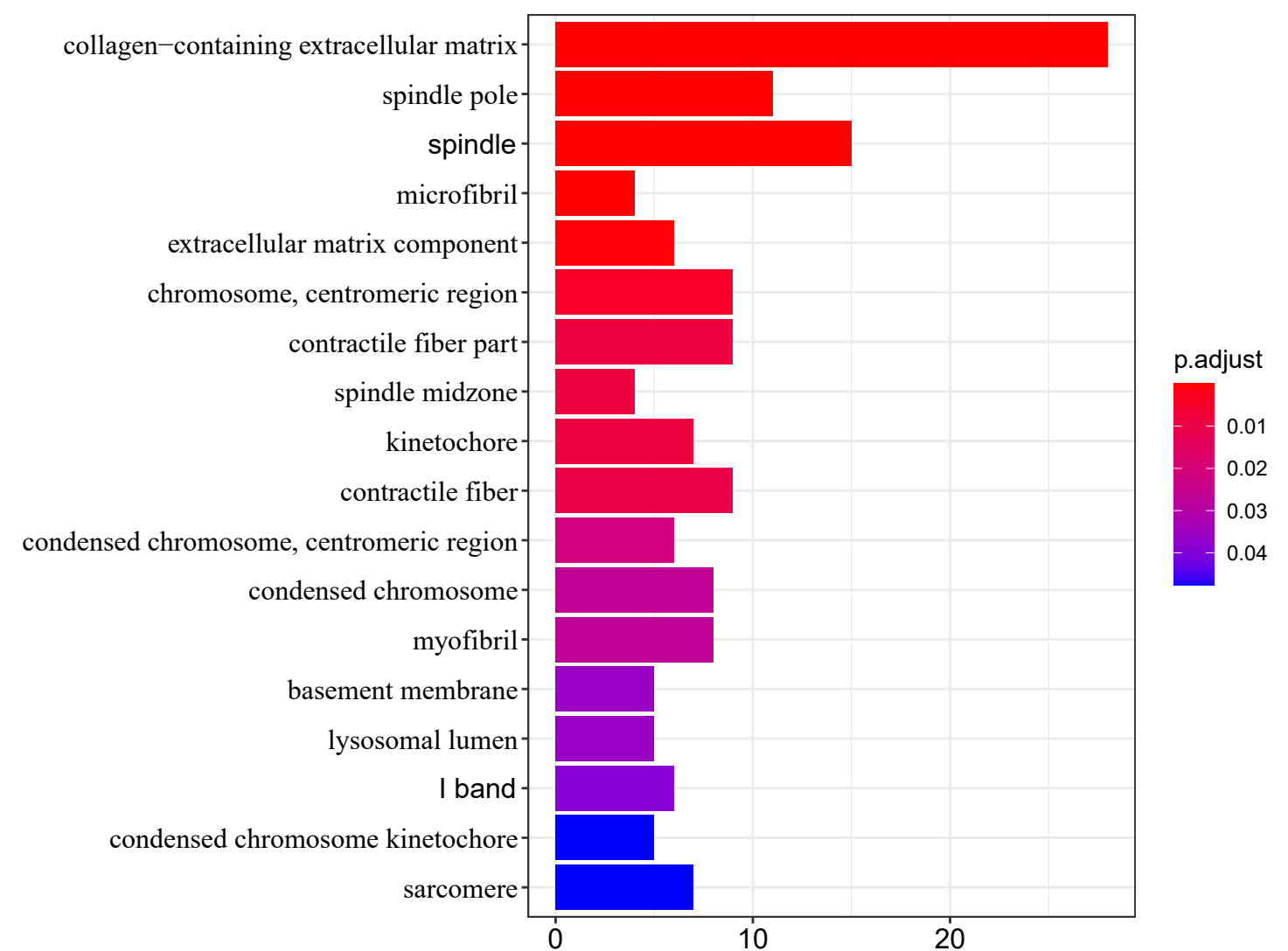

C

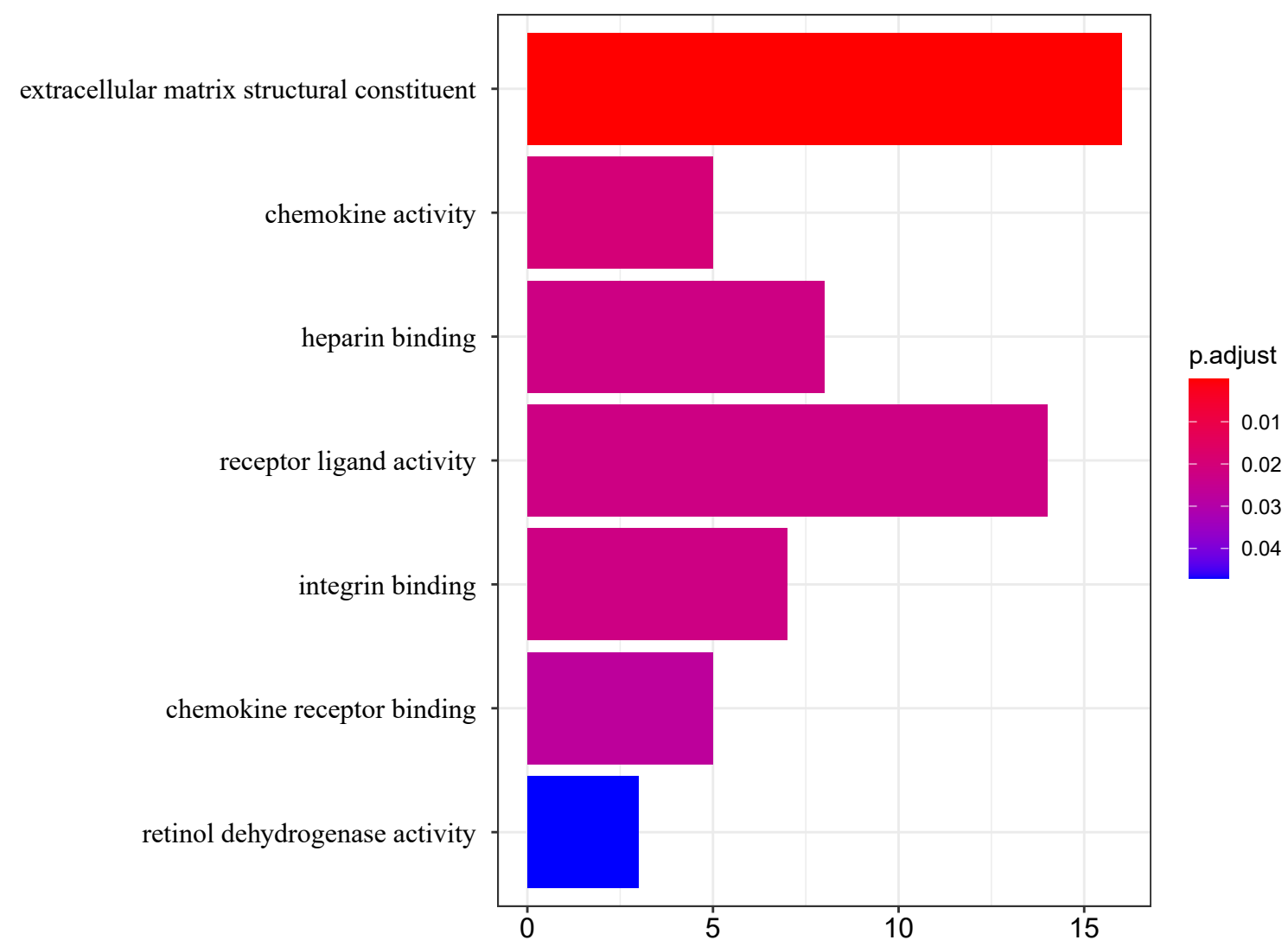

D

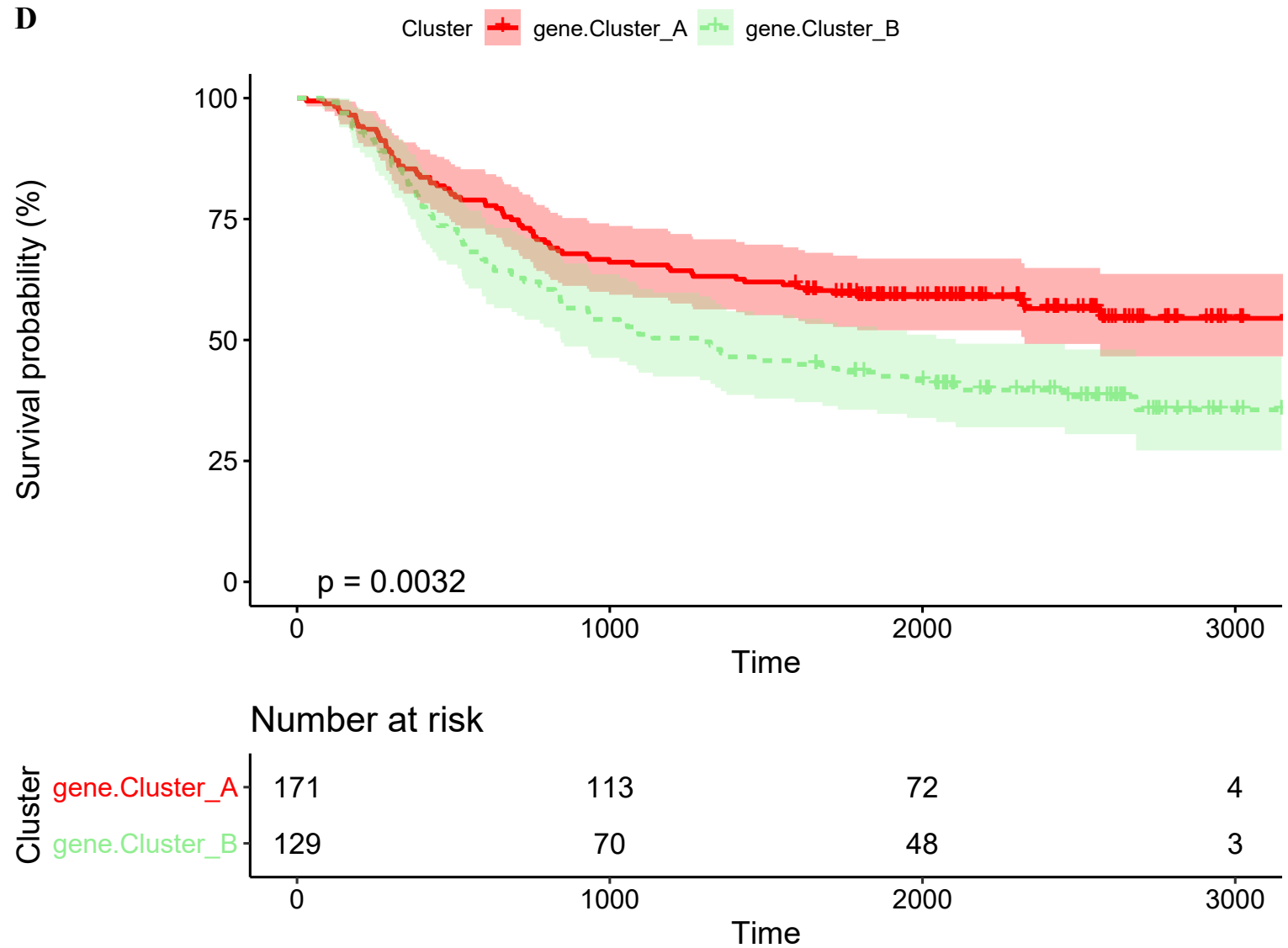

Supplement: Supplementary file 7 — Additional file 7: Fig. S7. Comparison of WM score and EMT score on hazard ratio, C-index, and 1-year, 3-year and 5-year AUC. CI, confidence interval. AUC, area under ROC curve. [file 40246_2022_386_MOESM7_ESM.pdf]

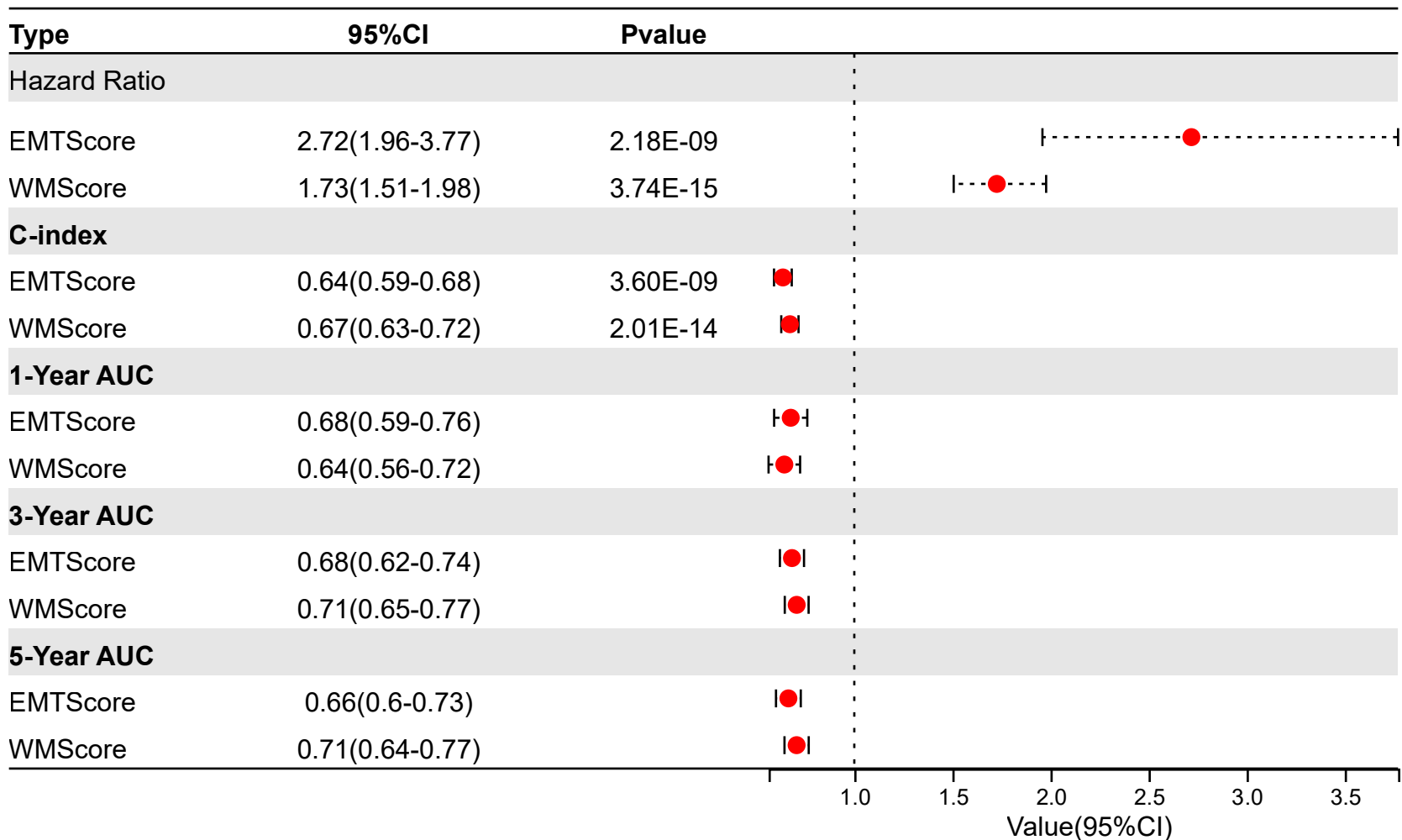

Supplement: Supplementary file 8 — Additional file 8: Fig. S8. Comparison of WM score and EMT score on hazard ratio, C-index, and 1-year, 3-year and 5-year AUC. CI, confidence interval. AUC, area under ROC curve. [file 40246_2022_386_MOESM8_ESM.pdf]
